# Automated Urban Travel Interpretation: A Bottom-up Approach for Trajectory Segmentation

**DOI:** 10.3390/s16111962

**Published:** 2016-11-23

**Authors:** Rahul Deb Das, Stephan Winter

**Affiliations:** Department of Infrastructure Engineering, The University of Melbourne, Parkville 3010, Australia; winter@unimelb.edu.au

**Keywords:** trajectory, trip, activity, GPS, IMU, context, temporal calculi, machine learning, MaaS

## Abstract

Understanding travel behavior is critical for an effective urban planning as well as for enabling various context-aware service provisions to support mobility as a service (MaaS). Both applications rely on the sensor traces generated by travellers’ smartphones. These traces can be used to interpret travel modes, both for generating automated travel diaries as well as for real-time travel mode detection. Current approaches segment a trajectory by certain criteria, e.g., drop in speed. However, these criteria are heuristic, and, thus, existing approaches are subjective and involve significant vagueness and uncertainty in activity transitions in space and time. Also, segmentation approaches are not suited for real time interpretation of open-ended segments, and cannot cope with the frequent gaps in the location traces. In order to address all these challenges a novel, state based *bottom-up approach* is proposed. This approach assumes a fixed atomic segment of a homogeneous state, instead of an event-based segment, and a progressive iteration until a new state is found. The research investigates how an atomic state-based approach can be developed in such a way that can work in real time, near-real time and offline mode and in different environmental conditions with their varying quality of sensor traces. The results show the proposed bottom-up model outperforms the existing event-based segmentation models in terms of adaptivity, flexibility, accuracy and richness in information delivery pertinent to automated travel behavior interpretation.

## 1. Introduction

Travel is an inevitable part of human life, required in order to perform an activity which is not possible at a current location. However, changing the perspective, travel itself can become an *activity* in its own right, and again changing perspective, travel can be conceived as a *sequence of activities*, each consisting of a segment travelled in a single mode. This paper focuses on the automatic interpretation of a travel as a sequence of activities, i.e., segments travelled in a single mode of travelling, from sensor traces collected on smartphones. In particular, the role of granularity will be highlighted (e.g., between *getting on board of a bus*, *taking the bus*, or *going to work*), along with the ambiguity about the mode that comes along with it [1]. The elementary trips of a travel are connected by transfers, at certain granularities. Travel can be mediated by moving objects in the form of different transport modes, but we explicitly include unsupported body movement (walking, running) as a mode. Since any mode is mixed with unrelated bodily movements, such as walking through a bus, taking a smartphone out of a pocket while sitting on a bus, or turning the head while cycling, the interpretation of sensor traces has to deal also with other noise than only from the sensor characteristics.

Identifying trips automatically is important for understanding the travel demand in a city, people’s movement behavior, modal preferences, route choice, patronage, and for enabling various customized services in the context of “Mobility as a Service” (MaaS) [2]. With the emergence of context-aware computing in the light of MaaS, there is a need to develop a real-time mobility-based activity detection framework (e.g., real-time mode detection model): Only real-time detection can provide context-aware customer services such as providing car drivers with congestion-related routing information while protecting bus passengers from this to happen. Another instance could be activating auto-answering on the smartphone for a car driver (in order to avoid distraction) while ensuring that passengers on a tram remain unrestricted.

In order to understand people’s travel activities, traditional methods rely on paper-based, telephonic or face-to-face travel survey techniques in order to generate travel diaries. A travel diary contains the mobility information of a person in terms of trips, with their start time, end time, origin, destination and transport mode(s). Since there is a time gap between the actual travel and the reporting of the travel, such a reconstruction process often involves under-reporting, miss-reporting, and potentially bias. More recently, GPS-based travel surveys have been explored that collect movement data in the form of time-stamped coordinates along the travelled route [3,4]. Early GPS assisted travel surveys were based on in-vehicle tracking [5,6,7], and only with the recent emergence of smartphones equipped with positioning and other location sensors along with inertial measuring units (IMU) it has been possible to continuously track an individual across any mode of travelling [8].

The remaining challenge is to automatically interpret these sensor traces for travel activities (single mode trips) and transfers. In the current state-of-the-art, a trajectory is top-down segmented based on some critical events (e.g., a drop in speed) and then activity states are detected for each segment [9,10]. However, segmenting a trajectory based on some heuristics is subjective and involves vagueness and uncertainty in activity transition in space and time, and thus, obscures the recognition and modelling of transfers. Prior work, discovering activities (including transfers) using clustering techniques, has to deal with clusters of any shape and any size, and hence comes with a significant uncertainty and ambiguity as to where a transfer begins and ends vis-á-vis a trip start and end. In contrast, the present work assumes that within a very fine grained space-time frame the activity state will remain same: A finer kernel involves less uncertainty than that of a longer segment, and the trip end of one segment becomes the trip start of the next segment. The common point in time defines a transfer precisely in space and time, and thus involves less ambiguity than that of a clustering-based approach. This paper hypothesizes that a state-based *bottom-up* approach is more adaptive than any top-down approach, and in addition will be able to detect activity states in a progressive manner (i.e., in near-real time). This translates into the temporal uncertainty depending on the length of space-time kernel. The shorter the kernel the less is the uncertainty, but at a cost of overall detection accuracy.

**Figure 1:**
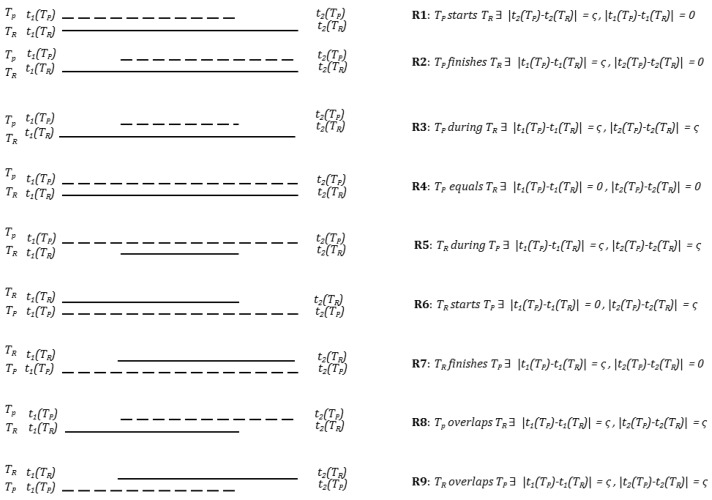
Trip uncertain temporal relationships between a reported trip ($T_{R}$) and predicted trip ($T_{P}$) based on Allen’s temporal calculus. In this figure $t_{1}$ and $t_{2}$ are the start time and end time of a given trip respectively.

In the state-based bottom up approach an atomic kernel is ran over the entire sensor trace and a particular activity state is detected iteratively. The assumption behind this approach is, shorter the temporal kernel more homogeneous the activity state will be. A transfer is then modelled with a given temporal uncertainty when there is a change in the activity state. The approach can be extended to a multi-grained atomic kernel approach to drill down the activity states, e.g., first detecting the travel activities, then the finer grained activity states during any transfer. The hypothesis has been tested on two different data sets: A trajectory data set of multi-modal inner-urban trips, and also a data set of inertial measurement unit (IMU) observations on-board a smartphone (without location information). The experiments prove that the new approach is not only more expressive in terms of richer travel information, but also capable of near-real time trip analysis as required for context-aware services. The contributions of this paper are as follows:

(a) Unlike the earlier approaches which are mostly behavior-based depend on a particular event(s) (say, drop in speed), this research presents a novel state-based bottom-up framework to segment the trajectories in a progressive way at different granularity.
(b) The aspect of temporal uncertainty in activity transition is explored and modelled using Allen’s temporal calculus [11]—which was missing in the earlier trajectory segmentation, trip generation and transport mode detection research (Figure 1).
(c) The framework presented in this paper is modular, adaptive, flexible and robust, and yet accurate. Since the framework uses an atomic kernel of definable length it can work in different granularities (e.g., for travel mode or transfer interpretation), and even in near-real time (defined by the kernel length). The framework can also handle varying data quality and richness in information content in the sensor trace.

Thus, in case of a top-down approach depending on a certain behavior or event, a trajectory is first segmented into a number of segments; and then an activity state is detected over each segment. On the other hand, for a bottom-up approach, a given activity state is detected within a short temporal kernel without considering any change in behavior of the moving object. Then the subsequent states are discovered iteratively, and a progressive segmentation takes place along the given trajectory.

The remainder of the paper is organized as follows. Section 2 gives an overview of the current state of knowledge and research gap. Section 3 defines some key concepts used in this research. Section 4 outlines the methodology used for data pre-processing and model building. Section 5 discusses the data sets, experiments and results. Section 6 reflects on the framework, which is followed by concluding remarks and an outlook in Section 7.

## 2. Literature Review

Travel diaries are a record of people’s travel history and also other activities performed during the travel. This kind of information is important for understanding people’s travel and activity behavior, which is considered an input for different types of travel demand models within a given space and time. In order to collect such travel diaries, travel surveys are conducted, which are currently paper-based or telephonic in most of the places in the world [12,13]. As surveys rely on people’s ability to recall their past travel behavior accurately and at different granularities this approach is generally subject to quality issues such as *under-reporting*—missing trips, missing transport mode annotation, missing transfers, unknown travel speeds—, *miss-reporting*—wrong transport mode annotation, inaccurate trip start and end times and locations—, and *bias*— deliberate manipulation of the provided information [14].

In order to overcome these issues GPS assisted travel surveys have been developed, with a first proof-of-concept run in Lexington, Kentucky [15,16,17]. GPS-based travel surveys can generate travel records in the form of a sequence of time ordered GPS points on the fly (trajectories) and thus are free from post-travel recall. However, the trajectories need to be interpreted as travel and other activities in order to fulfil the requirements of a household travel survey.

The efficacy of GPS-based surveys has proven that such in-situ sampled space-time information produces higher quality data than that of a paper-based travel diary [18,19,20]. For the trajectory analysis generally a clustering-based approach is used to detect trip start and end times. However, GPS trajectories are subject to discontinuity due to signal loss, for example, in urban canyons, under dense foliage, in buildings, or when travelling underground. Signal loss produces semantic gaps in clustering-based approaches, and thus creates false origins or destinations. Especially trip end detection—i.e., the end of a single mode travel segment—and trip purpose identification are of interest [7]. The trip end is detected based on a longer period of non-movement or longer dwell time at a given location. Prior studies suggested a threshold of 120 s to detect a trip end [7,18,21,22,23]. Stopher and others detected a trip end using a rule-based algorithm that considers trip characteristics before and after possible GPS signal gaps [24,25]. In order to detect the trip purpose Wolf developed a ‘point-in-polygon’ approach that uses a number of pre-defined land use types and trip purpose classes and evaluates if a trip end falls within a given land use type [6,7].

With the advancement of information and communication technology (ICT) and miniaturization of location and IMU sensors on-board mobile phones, it has been possible to obtain travel and other activity information in the form of trajectories without burden, and across any mode of travelling and any form of environment [26]. Asakura and Hato conducted a first mobile phone-based pilot survey in Japan [27]. Following that, Ohmori and colleagues developed another mobile phone-based travel survey application with a manual intervention, with 10 min sampling intervals [28]. Itsubo and Hato conducted a mobile phone-based travel survey on 31 respondents over five days with 30 s sampling intervals [29]. However, the earlier mobile phone-based surveys require significant amount of user intervention and offer limited flexibility in phone usage and sampling rates, and thus the users cannot follow their true travel behavior. With recent emergence of smartphones researchers started coming up with more user-friendly applications. These smartphone-based travel survey techniques can generate high quality travel and activity information in the form of trajectories generated from GPS, Wi-Fi and 3G/4G localization, combined with traces from IMU. These raw trajectories and sensor traces reveal location information and geometric patterns of the user movements [30,31,32]. In order to interpret the trajectories semantically, i.e., with regard of trips, travel modes, and activities, additional information relevant to the context and application domain is associated with raw trajectories [33]. For this purpose, the trajectory is segmented into a number of segments where each segment is analyzed to detect a given activity state (e.g., transport mode) or kinematic behavior (e.g., travel speed).

Gonzalez and colleagues developed a smartphone-based travel survey and detected transport modes using a neural network model [34]. Charlton and colleagues developed a similar application but focused on bicycle travel behavior [35]. Recently, Cottrill and colleagues developed the Future Mobility Survey (FMS), a full-scale household travel survey that consists of four phases: registration, pre-survey, activity diary, and a follow-up feedback survey [8,36]. In the registration phase the participant registers on the FMS portal providing basic household information. In the pre-survey more information on the socio-demographic profile of the household members is provided. In the activity diary phase the actual travel behavior is recorded, and recorded trajectories are uploaded to the FMS platform for a backend mode detection model. Finally, the inferred trips, modes and activities are validated by the participants in the feedback survey, a web-based prompted recall survey [37]. Similar applications have been developed elsewhere [38] for detecting transport modes from smartphone trajectories.

In order to analyse the trajectories segmentation is performed. Spaccapietra and colleagues developed an episodic algorithm known as stop-and-move-on-trajectories (SMoT) from a top-down perspective: first the trajectory is segmented into a number of segments and then an activity state is detected over a particular segment using a machine learning approach or expert system-based model [39]. This algorithm assumes a person will stop at a certain location for minimal amount of time in order to perform a certain activity and then start moving until reaching the next destination. Thus a raw trajectory is segmented into two different episodes and each episode is semantically enriched. A move episode reflects a person’s travel behavior, whereas a stop episode reveals a person’s activity behavior within a constrained space.

The SMoT algorithm was implemented in different forms. Alvares and colleagues developed an intersection-based approach (IB-SMoT) to model the stop and move episodes. IB-SMoT evaluates which spatio-temporal points of the trajectory intersect a given candidate region for a minimal time duration [30]. If the respective points satisfy the spatio-temporal condition those points will be considered as stop points, and the points that do not fall within a candidate region will be considered as move points. Palma and colleagues developed clustering-based stops and moves (CB-SMoT) where a clustering kernel is run over a trajectory, and the clusters containing low speed points with respect to a predefined threshold are called potential stop clusters [40]. Then each potential stop cluster is investigated if the cluster intersects any given region of interest and labeled as stop episode. Das and colleagues developed a density-based clustering algorithm based on the CB-SMoT approach but considering the speed, temporal duration and the proximity to nearest points of interests (POI) in order to detect the transfers [41]. However, a density-based clusters can be of any shape and any size and hence the crisp activity (or trip) start and end is ambiguous. Following the same line, Rocha and colleagues developed a direction-based algorithm (DB-SMoT) based on change of directions of GPS points in a trajectory [42]. Ashbrook and Starner developed a predefined clustering method to detect the stops from GPS trajectories [42]. On the other hand, Zimmermann and colleagues developed a spatio-temporal clustering method to detect the stops and moves [43]. In the same line, Andrienko and colleagues developed a stop detection framework by considering temporal duration and a user defined distance threshold [44]. Gong and colleagues extended the traditional clustering based stop detection approach by incorporating a machine learning module. They have developed a two stage model for detecting stops and stop types. Gong and colleagues used an improved clustering algorithm (C-DBSCAN) to detect the stops based on the spatial proximity of the GPS points. Then they have used a SVM-based supervised machine learning technique to infer the stop type in terms of activity or non-activity [45]. However clustering-based approaches work well on the dense GPS trajectories with good to moderate positional accuracy. During signal gap or in urban canyon clustering-based approach does not work well.

Assuming walking is necessary between two non-walking episodes, Zheng and colleagues proposed a walking-based segmentation approach in their transport mode detection research [10]. Once the entire trajectory is segmented based on speed profile (potential walking episodes), other non-walking segments are fed into a number of machine learning models and a transport mode is detected. Zheng and colleagues used four popular machine learning models: decision tree (DT), Bayesian network (BN), conditional random fields (CRF) and support vector machine (SVM), with highest accuracy of 75% using a DT model. Zheng and colleagues used five kinematic features and four modal classes. Stenneth and colleagues developed another decision tree-based predictive model with 93.5% accuracy [46]. A similar approach (walking-based segmentation) was adopted by Biljecki and colleagues who developed a Sugeno type fuzzy logic-based transport mode detection model with 91.6% accuracy [47]. Xu and colleagues developed a similar fuzzy logic-based model that can detect four different modalities with 93.7% accuracy by adopting a walking-based segmentation approach [48]. Yang and colleagues developed a two-stage approach for detecting modes with a core focus on distinguishing bus and car modality on a set of trajectories collected by handheld GPS devices [14]. In the first stage a machine learning algorithm was used to distinguish walk, bicycle and motorized trips. Then in the second stage a motorized mode is further identified as bus or car using a critical point method [14]. Mountain and Raper used a change in speed and direction for segmenting a trajectory [49]. However, a low speed (or walking) based segmentation approach creates ambiguity in certain cases especially when a vehicle moves slowly in heavy traffic or due to bad weather condition. Xia and colleagues proposed a GPS and accelerometer-based model with 50 Hz sampling frequency without any walking-based or clustering-based segmentation approach. Xia and colleagues detected four activity states such as stationary, walking, bicycle, motorized modes using a SVM with 96.3% accuracy [50].

However, such top-down segmentation approaches first segment the trajectory based on either a stop episode or a low speed or walking episode, and then attempt to detect a particular activity state or travel behavior over other segments. But as mentioned in Section 1, this approach is subjective and creates spatial and temporal ambiguity [51], and thus, if each of the segments is viewed as a specific trip then there is an uncertainty (or misalignment) of trip start and end (from segmentation perspective) and uncertainty of activity state (e.g., transport mode) along a given trip (from activity detection perspective). A vast majority of literature on transport mode detection and trip generation does not address this ambiguity during the trajectory inference process. Recently, from the transport mode detection perspective Prelipcean and colleagues developed a new error measure based on the quality of alignment of inferred segment to their ground truth counterpart to address such uncertainty during segmentation based on Allen’s temporal calculi [52]. Prelipcean and colleagues modeled three types of error measures using a cardinality of the measurement and spatial and temporal discrepancy such as implicit, explicit-holistic, and explicit-consensus-based segmentation [52]. However, their framework is limited and cannot model all the possible temporal relations in the context of trips. In this paper we have figured out four different types of trips that may possible during a single mobility-based action and their temporal inter-relationship.

In order to detect mobility-based activities in real time other researchers developed a temporal window-based approach. Hemminki developed an accelerometer-based transport mode detection model with a 1.2 s time window that can produce 84.2% accuracy [53]. Reddy and colleagues integrated an accelerometer and a GPS sensor to detect the modality in 1 seconds and achieved 74% accuracy [54]. Byon and colleagues used comparatively higher time window (10 min) to detect modalities without segmenting the GPS trajectories [55].

A similar approach has also been developed in detecting micro-level activities involved with body parts movement or small scale locomotion in an indoor environment. Such micro-level activities are known as activities of daily living (ADL) such as running, walking, jogging, brushing teeth, talking on phone, hand washing and performing various travel related actions—to name a few [56,57,58]. These activities are usually detected based on sensors, especially the CCTV installed in the environment and then analyzing the still images or video scenes [59]. With the emergence of IMUs on smartphones now such micro-level activities are easily detected almost everywhere in offline as well online mode [60,61].

Thus the existing research in real time urban transport mode detection as well as most of the activity recognition research in public health and mobile computing attempt to detect the activity within a queried time window and do not attempt to model the uncertainty of the continuity of a given activity. That means, the existing activity recognition research lacks in providing the information on activity start and end.

In this research we use the existing temporal window (kernel)-based approach (but with an introduction of iterative temporal merging) in order to detect an activity in real to near real time as well as detecting activity transition at different granularity using different sensor combinations. Thus in contrast to the existing real time approaches [53,54,55], where a time window is given and an activity state is to be detected, we have extended that approach and can detect an activity within a given time window as well, and given an activity the model can detect its start time and end time. In contrast to the offline approaches on trajectories where a subjective segmentation is performed, this paper presents a simple yet effective approach for segmenting the trajectory based on activity states in a fine grained time window. We also investigate how activity detection accuracy varies with different sensor combinations and different feature types.

## 3. Preliminaries

In this section, we will present some key definitions and basic theory behind the proposed adaptive travel interpretation framework.

### 3.1. Definitions

#### 3.1.1. Travel

Travel is a phenomenon of moving from one location to another location over time. Travel can be viewed as an *activity*—a temporally extended process—or an *action*—a not further expanded event—depending on the context of the travel analysis [1]. Furthermore, this notion of travel is open across a range of spatial scales. Inner-urban travel happens generally at environmental scale [62], but single parts, such as transfers between modes, can happen in vista scale. Inter-city travel is travel on geographic scale. In this research we are mainly interested in urban travel.

#### 3.1.2. Sensor Trace (Γ)

A sensor trace is a time ordered set of sensor observations that capture a user’s activity state at a specific granularity defined by the sampling frequency. In this research the sensors are assumed to be installed on a smartphone, and may include a location sensor as well as an inertial measurement unit. A sensor trace Γ consists of the signals of one or more sensors $I_{i}$ (including sensors operating on different channels), i∈[1,n], each expressed as a set of {s(k)} where k∈[1,m] and *m* is an integer. A sensor trace can be mathematically expressed as:

(1) $$\Gamma =\left\{I_{i}\right\}:I_{i}=\left\{s{\left(1\right)}_{i},......,s{\left(m\right)}_{i},t_{i}\right\}|\forall i:t_{i-1}<t_{i},\;i\in \left[1,n\right]$$

#### 3.1.3. Trajectory (Π)

A trajectory is a sequence of time ordered spatio-temporal points that represent a person’s travel history with coordinates in a three-dimensional Euclidean space ($x_{i},y_{i},z_{i}$) at a given time ($t_{i}$). In this research the ‘z’ value will be ignored as not relevant. However, a ‘z’ value can be integrated where the altitude information is vital, for example, travels between levels of a complex built environment. From the definition of a sensor trace, all the trajectories that are captured using GPS sensors, WiFi or 3G/4G localization onboard a smartphone are a type of sensor trace. A trajectory can be mathematically expressed as follows:

(2) $$\Pi =\left\{P_{i}\right\}:P_{i}=\left(x_{i},y_{i},\left[z_{i}\right],t_{i}\right)|\forall i:t_{i-1}<t_{i}$$

Depending on the information content and level of processing a trajectory can be classified into three distinct types as follows.

- *Raw Trajectory ($\Pi_{R}$)*: A *raw trajectory* is an unprocessed set of time ordered spatio-temporal points with varying levels of inaccuracy due to the noise present in the sensor signals, or signal gaps.
- *Pre-processed Trajectory ($\Pi_{P}$)*: A *pre-processed* trajectory is a set of time ordered spatio-temporal points which is pre-processed and filtered to some extent in order to discard inaccurate spatio-temporal points and other noise present in the data set. The level of processing depends on the application context.
- *Semantic Trajectory ($\Pi_{S}$)*: Both raw and pre-processed trajectories suffer from a semantic gap between the movement history of the traveller and their movement behavior. Such a semantic gap can be bridged by enriching a raw or pre-processed trajectory by domain information including spatial, non-spatial and temporal information. A *semantic trajectory* is constructed from a raw trajectory through a semantic enrichment operation.

#### 3.1.4. Segment (*Seg*)

A segment is a connected sequence of a sensor trace between a defined start and end point in time. A segment must include a portion of GPS trajectory, and may include observations of the sensors on an IMU.

#### 3.1.5. Atomic Segment (*ASeg*)

An atomic segment is the smallest segment of a sensor trace, defined by a context-dependent kernel length.

#### 3.1.6. Atomic Kernel ($K_{\eta }$)

**Figure 2:**
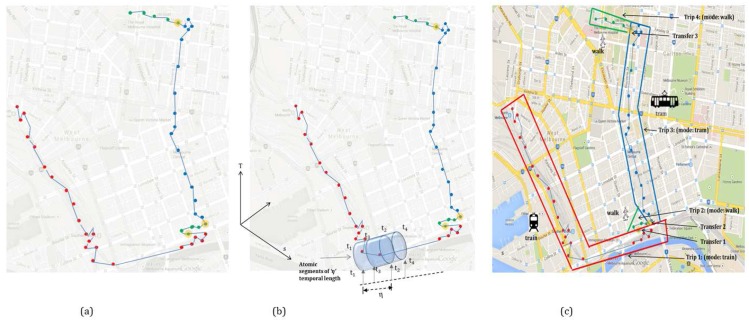
A raw trajectory is shown in Figure (**a**); Atomic segments are generated using an atomic kernel of time length *η* on the raw trajectory in Figure (**b**); Using a state-based bottom-up approach a given trajectory is then segmented into four segments that are detected as four distinct trips based on different transport modes with three transfers in Figure (**c**).

An atomic kernel is an operator that extracts an atomic segment of a sensor trace, including a GPS trajectory. An atomic kernel has a defined, constant length (*η*) within a specific context (Figure 2).

#### 3.1.7. Trip (*T*)

A trip is an action of changing location with a purpose. A travel can consist of more than one trip. A trip is characterized by a constant transport mode. Thus, the trips are attributed by their start location and time, end location and time, and a given transport mode. There may be different types of trips:

- *Actual Trip ($T_{A}$)*: An actual trip is what happens in reality while traveling from one location to another location.
- *Reported Trip ($T_{R}$)*: A reported trip is the trip that is annotated or reported by the traveler from memory, which often involves quality and granularity issues.
- *Scheduled Trip ($T_{S}$)*: A scheduled trip is a trip that is predefined by a given transport service with its trip origin, destination, trip start time, end time, route and the intermediate stops that are to be visited along during the travel.
- *Predicted Trip ($T_{P}$)*: A predicted trip is a trip that is inferred from a predictive framework based on the features computed from a given sensor trace that may include a GPS trajectory and IMU information.

#### 3.1.8. Transfer (*Trans*)

A transfer is an action of changing from one transport mode to another transport mode.

#### 3.1.9. Transport Mode (*M*)

A transport mode is a mediation of mobility, either by locomotion or by some vehicle. In the following experiment data was collected for four public transport modes: *bus*, *train*, *tram*, and *walk*. Other modes of urban mobility are *cycling*, *driving* a car, or *riding* in a car (being passenger in a car).

### 3.2. Uncertainties in Trips

Trips are characterized by start and end time locations, and a travel mode. Each of these characteristics can vary between the reported trip, the scheduled trip, the actual trip, and the predicted trip, leading to temporal, spatial and semantic uncertainties. These uncertainties may occur due to synchronization problems between clocks (such as the smartphone’s and the transport provider’s) or the memory or attention of the traveller when reporting a trip. The uncertainties also stem from the varied ontological commitments and cognitive perceptions of trip starts and transitions in actions (e.g., resolving a transfer into subsequent actions). The uncertainty can also arise from actual travel times other than the scheduled time, wrong inferences from the predictive model on the mode, and also uncertainties in sensor signal information (e.g., signal loss or multipath effects in case of a GPS trajectory).

### 3.3. Trip Uncertain Temporal Relationships

The experiment below is designed with trips reported in-situ, not from memory in hindsight. These reported trips will form the ground truth in the experiment, i.e., they are assumed to be correct representations of the actual trips. In this case it is difficult to model the temporal uncertainty between the reported trip and actual trip, although it must exist. Temporal deviations will also occur between the reported trip and the predicted and the scheduled trip. Such temporal uncertainties can be modeled qualitatively by using Allen’s interval calculus [11]. Figure 1 shows nine possible relationships between a predicted trip ($T_{P}$) and a reported trip ($T_{R}$) where ς is the crisp uncertainty for time observations predefined at a given context. It will be shown later how the inference accuracy varies by varying the ς value. The relationships also hold between a reported trip ($T_{R}$) and a scheduled trip ($T_{S}$), or a scheduled trip ($T_{S}$) and a predicted trip ($T_{P}$).

### 3.4. Predictive Model

**Figure 3:**
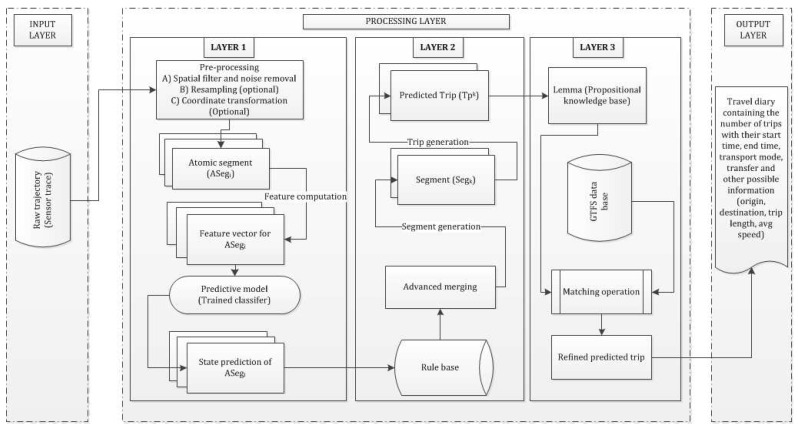
A state-based bottom-up framework for travel dairy generation.

A predictive model is a module in this framework in Layer 1 in the processing phase (Figure 3) that detects a given activity state. A predictive model is basically a classifier constructed based on a number of features (Section 4.3.4). In this paper a number of machine learning algorithms have been investigated to construct the best predictive model in Layer 1 which is explained in Section 5.

## 4. Trajectory Segmentation Frameworks

In this section we will present the existing trajectory segmentation frameworks that detect the trips based on different criteria (see Section 2). We then present our novel state-based trip detection framework, which detects the trips more adaptively along with rich behavioral information (e.g., transport mode state).

A trip can be modeled as a particular segment with a homogeneous state and distinct behavior. Trajectory segmentation approaches can be classified into two broad categories: behavior-based and state-based approaches. Behavior-based approaches segment a trajectory into meaningful parts and then infer a state for each segment. Thus, these approaches are top-down. The number and type of segmentation operations is context dependent. In contrast, the state-based approach developed in this paper extracts an atomic segment assuming the state will remain constant in that fine grain, and then the state is detected using a hybrid approach (machine learning and heuristic rules), whereupon homogenous segments are generated using an advanced merging operation, which will generate the trips. Thus, the second approach is richer in information content and more adaptive. This approach is bottom-up. This paper presents the novel state-based bottom-up approach, which is compared with the two state-of-the-art top-down approaches: a walking-based approach and a clustering-based approach (and its variants), which is basically a realization of the SMoT algorithm [30,39].

### 4.1. Trip Detection by a Walking-Based Approach

A walking-based approach is a variant of the behavior-based approaches where the behavior is attributed to drop in speed. It is generally used in order to segment a trajectory in the context of transport mode detection.

The assumption behind a walking-based approach is that people need to walk in between two different transport modes [10]. In this regard, a walking segment is detected by deterministic rules where the key parameters are speed ($\frac{dl}{dt}$), merging distance (δl), and total distance (L) traveled over a segment. However, by relying on these parameters this approach is subjective and thus it is difficult to set the threshold parameters.

Since a GPS trajectory is prone to signal loss and multipath effect a walking-based approach needs a thorough pre-processing of the raw trajectory. The trajectory is filtered in such a way that no high speed points remain in between two low speed points and vice-versa. The filtering process should also remove points with high DOP values (or spatial uncertainties). In this research the speed threshold is considered 9 km/hr based on prior research [63], and the merging distance is 20 m based on trial and error. The total distance threshold for a segment to qualify as a walking segment is iteratively tested from 10 m to 200 m. Algorithm 1 presents a two stage pre-processing operation where a GPS trajectory is first filtered based on spatial uncertainty (Spatial_Filter) followed by speed outliers (Velocity_Filter). The walking-based technique is then presented in Algorithm 2.

**Algorithm 1** Pre-processing of a GPS trajectory in two stages  1:**INPUT** 1)$\Pi_{R}:$ rawTlist(), 2)lowSpeed: LST  2:**OUPUT** $\Pi_{P}\;[\mathrm{spatialFiltered}:\;\mathrm{sfTlist}();\;\mathrm{velocityFiltered}:\mathrm{vfTlist}()]$  3:**PROCEDURE Spatial_Filter()**  4:$rawTlist.size\left(\right)=k_{1}$  5:**for** i=0 to $k_{1}$−1 **do**  6:    **if** rawTlist.get(i).getAccuracy()>40 **then**  7:        $sfTlist.add(P_{i})$      # rawTlist.get(i)=$P_{i}$, where $P_{i}$ is a GPS point in raw trajectory $\Pi_{R}$  8:    **end** **if**  9:**end** **for**10:**END PROCEDURE**11:**PROCEDURE Velocity_Filter()**12:$sfTlist.size\left(\right)=k_{2}$13:**for** i=1 to $k_{2}$−1 **do**14:    **if** sfTlist.get(i−1).getSpeed()>LST||sfTlist.get(i+1).getSpeed()>LST **then**15:        vfTlist.add($P_{i}$)16:    **else**17:        **if** sfTlist.get(i).getSpeed()<LST **then**18:            **if** sfTlist.get(i−1).getSpeed()≤LST||sfTlist.get(i+1).getSpeed()≤LST **then**19:                   vfTlist.add($P_{i}$)20:              **end** **if**21:        **end** **if**22:    **end** **if**23:**end** **for**24:**END PROCEDURE**


**Algorithm 2** Trip generation using walking-based approach  1:**INPUT** 1)$\Pi_{P}:$ pTlist(), 2)lowSpeed: LST, 3)mergingDistance: δl, 4)totalDistance: L  2:**OUPUT** a set of trips  3:**PROCEDURE Trajectory_Segmentation()**  4:$pTlist.size\left(\right)=k_{3}$  5:**for** i=0 to $k_{3}$−1 **do**  6:  **if** pTlist.get(i).getSpeed()≤LST **then**  7:      templist.add($P_{i}$)  8:  **else**  9:      **if** pTlist.get(i).getSpeed()>LST **then**10:            **if** templist.size()>0 **then**11:                    seglist.add(newlist(templist))12:                    templist.clear()13:            **end** **if**14:      **end** **if**15:  **end** **if**16:**end** **for**17:**END PROCEDURE**18:**PROCEDURE getPotential_Walking_Segments()**19:**if** seglist.hasMergeableSegments(δl) **then**20:  mergedSeglist=segmentMerging(seglist)               #   merging all the mergeable short low speed segments21:**end** **if**22:$mergedSeglist.size\left(\right)=k_{5}$23:**for** i=0 to $k_{5}$−1 **do**24:  **if** mergedSeglist.get(i).getLength≥L **then**25:    walkingSeglist.add(mergedSeglist.get(i)            #    walking segments are detected26:  **end** **if**27:**end** **for**28:nonWalkingSeglist=getNonWalkSegments(walkingSeglist)  #non walking segments are extracted29:**END PROCEDURE**


### 4.2. Trip Detection Based on Clustering-Based Approach

A clustering-based technique is another popular approach for trajectory segmentation. Since a clustering technique is based on proximity of GPS points the clusters generated over a trajectory bear a semantic significance, for example, where the traveller has been stationary or had limited body movement for a certain time period. The notion behind a clustering-based approach is that during traveling on different modes people transfer or do some static activity (say, in a station, office, or home). In this research the clusters are assumed as the extent in space and time where a transfer takes place in order to change from one transport mode to another.

In this paper, a clustering-based algorithm is implemented using a spatial clustering application with noise (DBSCAN). DBSCAN is initialized with an arbitrary point ($P_{i}$) in the trajectory. The algorithm then searches for neighbor points (N) within an *ϵ*-neighborhood of point $P_{i}$. If N ≥ minPts then $P_{i}$ is defined as core point. The parameter ‘minPts’ is the minimum number of points to be present in the neighborhood of any given point in order to qualify that point as a core point. The algorithm then evaluates the next point and grows the cluster(s) until all the points are visited.

Once the clustering operation is performed there may be a number of clusters of different shape and size. In order to extract the most potent clusters (in the context of trip detection) a merging operation is performed followed by a relevance measure check. The merging operation is performed based on inter-cluster spatial distance threshold (ICSD) and inter-cluster temporal duration threshold (ICTD). However, a spatial clustering may raise the risk of clustering the to and from points together and thus leading to erroneous trip modeling. In order to deal with this issue a temporal proximity (tdiff) is used along with the spatial proximity (*ϵ*) to modify the basic DBSCAN into spatio-temporal DBSCAN (ST-DBSCAN).

However, there may also be some clusters that form without characterizing a transfer, for example, due to vehicle stops for pickup or drop-off or at traffic lights, or over a walking trip where the speed of walking is low, such as a stroll in a park or moving in a crowd. In order to filter such irrelevant clusters a temporal relevance check is performed over all the clusters. If the duration (Φ) of the cluster is greater than or equals to a temporal threshold then that cluster qualifies as a relevant cluster or a potential transfer zone. That said, clusters can be of any shape and size and hence from ontological point of view it is difficult to model the trips with their start and end in space and time. Algorithm 3 demonstrates a spatio-temporal clustering on trajectories to retrieve the transfer information.

**Algorithm 3** Spatio-temporal clustering on trajectories
  1:**INPUT** 1)$\Pi_{R}:$ rawTlist(), 2)Neighbors: minPts, 3)search radius: *ϵ*,  2:**INPUT** 4)temporal proximity: tdiff, 5)ICSD, 6)ICTD  3:**OUPUT**a set of clusters denoting possible transfers in a trajectory $\Pi_{R}$  4:**PROCEDURE ST_Clustering()**  5:clusterlist=getST_DBSCAN (rawTlist, minPts, *ϵ*, tdiff)  6:clusterlist.size()=$k_{1}$  7:**for** i=0 to $k_{1}$−1 **do**  8:  **if** spatialDistance(clusterlist.get(i),clusterlist.get(i+1))≤ICSD **then**  9:      **if** temporalDistance(clusterlist.get(i),clusterlist.get(i+1))≤ICTD **then**10:            $cluster_{i}=merge\left(clusterlist.get\left(i\right),clusterlist.get\left(i+1\right)\right)$11:            clusterlist.remove(i,i+1)12:            clusterlist.add($cluster_{i}$)13:      **end** **if**14:  **end** **if**15:**end** **for**16:clusterlist.size()=$K_{2}$17:**for** i=0 to $K_{2}$−1 **do**18:  **if** clusterlist.get(i).getDuration≥Φ **then**19:      $cluster_{i}=clusterlist.get\left(i\right)$20:      transferlist.add($cluster_{i}$)21:  **end** **if**22:**end** **for**23:**END PROCEDURE**


In this paper two existing top-down approaches (walking-based and clustering-based) have been implemented along with the proposed state-based approach for a comparative study. Although there could be different variations of the two above mentioned top-down approaches (see Section 2), but in general such approaches are inadequate in certain circumstances. For example, the walking-based approach requires a consistent and good quality GPS signal, it cannot handle IMU information, and it completely fails when there is no GPS signal for a prolonged period of time. On the other hand the clustering-based approach is robust to GPS noise, but also cannot deal with the IMU information, and does not work very well on sparse GPS trajectory data. More significantly, the clustering-based approach suffers from the ontological ambiguity about start and end of a trip.

### 4.3. Trip Detection Using a State-Based Bottom-up Approach

In order to address these issues a more robust and adaptive state-based bottom up approach is proposed. The proposed approach can handle GPS noise as well as IMU information. The proposed approach is less subjective than the walking-based approach, and at the same time tends to generate activity transitions with a clear provision of trip start and end, which is missing in the clustering-based approach. A state-based bottom-up approach also generates rich activity information (in terms of transport mode along a given trip) and thus this proposed approach is more effective in terms of generating travel diaries at different granularity with different level of uncertainty.

A state-based bottom-up approach is a hierarchical framework consisting of three layers (Figure 3). The first layer is the input layer where a raw trajectory is fed in. The second layer is the processing layer that consists of further three sub-layers (LAYER 1, LAYER 2, LAYER 3), where the third layer is the output layer that generates the travel diary containing the trip information. In the first processing layer (LAYER 1) an atomic kernel is ran over the trajectory based on the query time that detects the activity states using a set of machine learning algorithms over each atomic segment. Thus, the first layer can also infer activity states (transport mode in this case) in near-real time. In the second processing layer (LAYER 2) an advanced merging operation is performed based on a set of heuristic rules. This will merge the consecutive atomic segments with similar activity states and predict the trips. In order to raise the confidence and strengthen the inference process, especially on trip start and trip end along with the transport mode used in that particular trip, a general transit feed specification (GTFS) information is used to evaluate the initial predicted trips in the third processing layer (LAYER 3). Figure 2 illustrates how the atomic kernel bounded in time [$t_{k-1}$, $t_{k}$] of duration ($t_{k}$−$t_{k-1}$=*η*∣k≥1) is ran over the trajectory and how different trips are inferred based on given transport modes. In the following section each layer is explained in detail. The model presented in this paper is a hybrid approach that leverages the machine learning algorithm(s) for the initial activity state prediction followed by processing the rule base.

#### 4.3.1. LAYER 1: Near-Real Time Activity State Detection

Since a trip is characterized by a set of time-ordered homogeneous sensor data points (that may include GPS data points), in the first layer a predictive model is developed that will detect the activity state based on a classifier. In order to train the classifier different types of kinematic and spatial features are computed using sensor signals.

In this context the *activity state* is traveling on a given transport mode, and the *transport mode* is the mediation of this activity. In order to detect the transport mode an atomic kernel is applied (with 50% overlap) over the trajectory (or sensor trace) to extract a set of atomic segments. Then a number of features are computed within each atomic segment and a feature vector is created. Thus, if there are *N* atomic segments then there will be *N* feature vectors for a given trajectory. In some literature the atomic kernel is termed as sliding window or sliding kernel. The overlap is necessary in order to capture all the possible kinematic behavior especially during state transition and sudden change in behavior. Once the feature vectors are generated training is performed on a number of classifiers. Then all the classifiers are evaluated using testing trajectories separately. Once all the atomic segments are inferred (from all the test trajectories) an advanced merging operation is performed to generate longer segments of homogeneous activity states, which will turn into predicted trips. The six machine learning-based classifiers are chosen based on prior research in transport mode detection and trajectory analysis.

#### 4.3.2. LAYER 2: Advanced Merging Operation and Potential Segment Generation for Trip Detection

In the second layer the atomic segments are merged sequentially based on similarity in their predicted transport mode (queried from Layer 1). The assumption behind such a merging operation is that all the points in a sensor trace or a portion of GPS trajectory that form a particular trip will bear a uniform activity state: traveling on one transport mode along one and the same trip from a given origin to a (temporary or final) destination. Thus, when there is a change in activity state, a new trip has started. The point in time and space where the transition occurs is the transfer point.

In the first stage in Layer 2 an initial merging is performed based on the initial transport mode inference. However, due to the diverse performance of classifiers and depending on the data quality and uncertainties in movement behavior there may be false positives. In order to address this issue a set of rules refines the merging operation of the consecutive atomic segments (Algorithm 4).

#### 4.3.3. LAYER 3: Trip Refinement Using GTFS and Spatial Information

**Figure 4:**
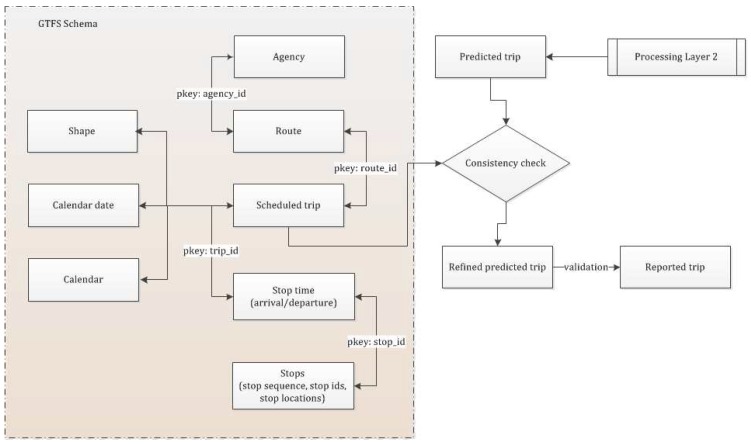
General transit feed specification (GTFS) schema and consistency check between the predicted trip and the scheduled trip.

Once the merged segments (predicted trips) are generated from Layer 2 a further refinement operation is performed using GTFS and other spatial information realizing the following five lemmas. In this layer a matching is carried out that matches the predicted trips with the scheduled trips based on spatial and temporal information along with the trip start and trip end with the scheduled stop information. For the following lemmas an $i^{th}$ predicted trip ($T_{P}$) is represented by a tuple of trip origin ($O_{p}^{i}$), trip destination ($D_{p}^{i}$), and predicted mode over trip *i* ($M_{p}^{i}\left(T_{i}\right)$). However, in order to match against the scheduled transit information the framework requires predicted stop and route information. Thus, a pair of stops at predicted trip origin ($S_{p}^{i}\left(O\right)$) and destination ($S_{p}^{i}\left(D\right)$) are queried using a variable search radius of 50 m, 100 m and 300 m progressively until at least one stop is retrieved around the trip origin point ($O_{p}^{i}$) and trip destination point ($D_{p}^{i}$). This information is then used to match the predicted trips with the scheduled trips and refine the prediction through the following lemmas. Figure 4 shows different component tables of GTFS schema with their common primary keys (*pkey*) and a consistency check between the predicted trip generated from Layer 2 in processing layer (with stop information at trip start and end) and a suitable scheduled trip, which is retrieved from the GTFS data.

**Algorithm 4** Rules for segment merging  1:**INPUT** All the atomic segments in a given trajectory (seglist), temporal threshold: Ψ  2:**OUPUT** a set of merged segments (mergedSeglist)  3:**PROCEDURE Segment_Merging()**  4:seglist.size()=k  5:boolean flag=false  6:**for** i=0 to *k*−2 **do**  7:  current_seg=seglist.get(i)  8:  next_seg=seglist.get(i+1)  9:  **RULE 1:**10:  **if** current_seg.mode_type==next_seg.mode_type&&next_seg.duration≤Ψ **then**11:      merge_seg=merging(current_seg,next_seg)12:      mergedSeglist.add(merge_seg)13:      flag=true14:  **end** **if**15:  **RULE 2:**16:  **if** current_seg.mode_type==next_seg.mode_type&&next_seg.duration>Ψ **then**17:      merge_seg=merging(current_seg,next_seg)18:      mergedSeglist.add(merge_seg)19:      flag=true20:  **end** **if**21:  **RULE 3:**22:  **if** current_seg.mode_type!=next_seg.mode_type&&next_seg.duration≤Ψ **then**23:      merge_seg=merging(current_seg,next_seg)24:      mergedSeglist.add(merge_seg)25:      flag=true26:  **end** **if**27:  **RULE 4:**28:  **if** flag==false **then**29:      mergedSeglist.add(current_seg)30:      mergedSeglist.add(next_seg)31:  **end** **if**32:**end** **for**33:**END PROCEDURE**

**Figure 5:**
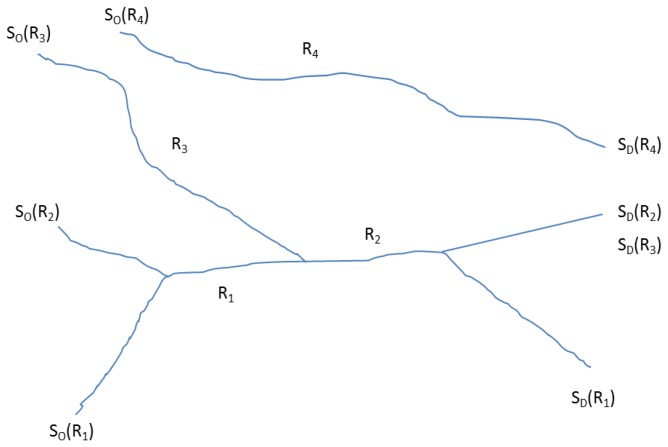
Some possible stop sequence ambiguity along different routes: $S_{O}\left(R_{i}\right)$ and $S_{D}\left(R_{i}\right)$ denote an origin and destination stop along route $R_{i}$. However, departure time at $S_{O}$ must be earlier than at $S_{D}$.

- **Lemma 1: Stop type similarity**
  Since a trip is a segment of the trajectory, which consists of sensor data points that bear the same transport mode state ($M_{p}^{i}$), the stops at trip start and end must be of type ($M_{p}^{i}$). For example if a trip is made by a tram then the start stop and end stop of this trip must be two tram stops.
  (3) $$M_{p}^{i}\left(S_{p}^{i}\left(O\right)\right)=M_{p}^{i}\left(S_{p}^{i}\left(D\right)\right)$$
- **Lemma 2: Disjoint stop relationship**
  Since the GPS signal is prone to multipath effects and occasional signal loss due to obstruction must be expected, not all GPS points are recorded, and instead of updates in the GPS feed successive points will be recorded as the last known point. However, technically the stop at the trip start and end must be spatially different if it is not a return trip.
  (4) $$S_{p}^{i}{\left(O\right)}_{l}\neq S_{p}^{i}{\left(D\right)}_{l}$$
  (5) $$\Rightarrow \left(X_{S_{p}^{i}\left(O\right)},Y_{S_{p}^{i}\left(O\right)},Z_{S_{p}^{i}\left(O\right)}\right)\neq \left(X_{S_{p}^{i}\left(D\right)},Y_{S_{p}^{i}\left(D\right)},Z_{S_{p}^{i}\left(D\right)}\right)$$
- **Lemma 3: Stop sequence (un)ambiguity**
  No pair of trip origin and destination stop may be the members of more than one scheduled trip. That said, two scheduled trips may have a portion of their routes overlapping with each other. There may also be two routes with the same pair of origin and destination stops but in reverse order (which is a typical case in a return travel along the same route but in different direction). In this case routes may overlap. Figure 5 illustrates some of these ambiguities in stop sequences for different routes.
  For the time being we will ignore the first case in lemma 3. In order to address the latter case the following proposition should be followed.
  **Proposition** **L3.1:** The end stop or destination stop ($S_{p}^{i}\left(D\right)$) should occur after start or origin stop ($S_{p}^{i}\left(O\right)$) in terms of time of visit (t).
  (6) $$S_{p}^{i}{\left(O\right)}_{t}>S_{p}^{i}{\left(D\right)}_{t}$$
- **Lemma 4: Closest time selection**
  The arrival and departure time at predicted origin and destination stops should be close to the scheduled stops in that location. However, there always exists a temporal uncertainty that makes the predicted trip start and end time deviate from the scheduled trip start and end time. For this purpose a temporal threshold (δt) is used. For origin stops this can be expressed as follows, and similarly for destination stops. This will also conform with the first case in Lemma 3.
  (7) $$|S_{p}^{i}{\left(O\right)}_{t}-S_{s}^{i}{\left(O\right)}_{t}|=\delta t$$
- **Lemma 5: Use of $WT_{i\pm 1}$ OD information**
  Due to signal loss and uncertainties in the inference process in Layer 1, some (predicted) non-walking trips may have wrong trip start and end time. And these trip origin and destination stops may not have any scheduled trips in common within a given temporal threshold (δt). To address this issue following proposition is made.
  **Proposition** **L5.1:** If there is no scheduled trip ($T_{S}$) found in the GTFS data base that matches a predicted trip *i* ($T_{P}^{i}$) in terms of the mode type (*M*) or temporal information (arrival/departure time) then the mode type ($M_{p}^{i}\left(S_{p}^{i-1}\left(D\right)\right)$) at the destination stop of the previous predicted trip ($T_{P}^{i-1}$) or mode type ($M_{p}^{i}\left(S_{p}^{i+1}\left(O\right)\right)$) at the origin stop of the next predicted trip ($T_{P}^{i+1}$) stops are considered, whichever is a walking trip (*WT*) in between $T_{P}^{i-1}$ and $T_{P}^{i+1}$.

The lemmata developed in this paper are not complete. Depending on the situation new lemmata can be added. However the lemmata presented in this paper are sufficient enough to deal with different spatio-temporal and predictive uncertainties of trip patterns. That said, the thresholds set to quantify the lemmata and length of the temporal kernel depend on the type of data quality, sampling frequency and mode types to be distinguished. For temporal kernels the length has been evaluated starting with the shortest possible duration depending on the sampling frequency. Empirically the length of the temporal kernel must be greater than the minimum sampling rate used to capture the sensor trace.

#### 4.3.4. Feature Computation for Detecting Near-Real Time Activity States in Layer 1

**Figure 6:**
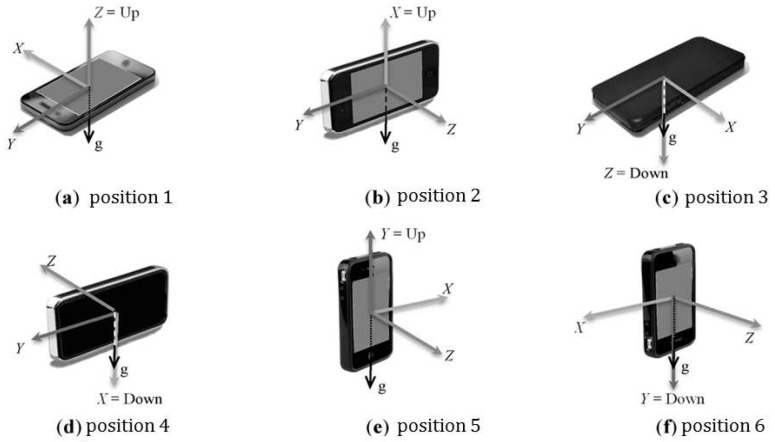
Smartphone axes in different directions. This figure has been reproduced from [64].

In order to construct the predictive model, a number of features are generated using different machine learning classifiers, inferring the activity state on a queried trajectory using a given kernel length (*η*) over $I_{n}$ number of data points. Three different case studies are presented (Context 1 Scenario 1, Context 1 Scenario 2, and Context 2) depending on the quality and granularity of data using different sensor combinations (e.g., GPS, 3-axis accelerometer, gyroscope and gravity sensor). Prior work has investigated the aspects of sensor calibration in the context of activity recognition [64]. However, in real to near-real time scenario the sensor information can come from different (unknown) smartphone sources owned by different users to a centralized server where the inference model is running. In such situation it is not always possible to get the hardware type, or mobile manufacturer information and thus poses difficulty in calibrating the particular source(s). To emulate the real world condition, thus in this paper no attempt has been made to calibrate the sensors. However, a low pass filter has been used to remove the noise present in the IMU signals used in the proposed framework (see Section 5). Figure 6 shows different axes of a smartphone in different positions [64]. Conventionally the axes will remain constant irrespective of the phone’s orientation.

A total of 34 features are computed using different sensor signals, based on acceleration in three directions such as (X: $A_{x}$, Y: $A_{y}$, Z: $A_{z}$), rotational vectors in three directions (X: $r_{x}$, Y: $r_{y}$, Z: $r_{z}$), pitch ($r_{x}$), yaw ($r_{z}$), roll ($r_{y}$), speed (*v*), and spatial proximity to the nearest route network using latitude, longitude information from a GPS sensor. In order to eliminate the gravity component a linear acceleration in three axes is chosen (X: $a_{x}$, Y: $a_{y}$, Z: $a_{z}$). The features generated are as follows:

- Average of linear acceleration in X-direction ($Avga_{x}$), Y-direction ($Avga_{y}$) and Z-direction ($Avga_{z}$)
  (8) $$Avga_{x}=\frac{\Sigma a_{x}}{I_{n}}$$
  (9) $$Avga_{y}=\frac{\Sigma a_{y}}{I_{n}}$$
  (10) $$Avga_{z}=\frac{\Sigma a_{z}}{I_{n}}$$
- Average of resultant linear acceleration ($Avga_{xyz}$)
  (11) $$Avga_{xyz}=\frac{\Sigma a_{xyz}}{I_{n}}$$
- Average of resultant rotational vector ($AvgR_{xyz}$)
  (12) $$AvgR_{xyz}=\frac{\Sigma r_{xyz}}{I_{n}}$$
- Average of rotational vectors in X-direction ($Avgr_{x}$), Y-direction ($Avgr_{y}$) and Z-direction ($Avgr_{z}$)
  (13) $$Avgr_{x}=\frac{\Sigma r_{x}}{I_{n}}$$
  (14) $$Avgr_{y}=\frac{\Sigma r_{y}}{I_{n}}$$
  (15) $$Avgr_{z}=\frac{\Sigma r_{z}}{I_{n}}$$
- Variance of linear acceleration in X-direction ($Vara_{x}$), Y-direction ($Vara_{y}$), Z-direction ($Vara_{z}$) and resultant linear acceleration ($Vara_{xyz}$)
  (16) $$Vara_{x}=\frac{1}{I_{n}-1}\sum {\left(a_{x}-Avga_{x}\right)}^{2}$$
  (17) $$Vara_{y}=\frac{1}{I_{n}-1}\sum {\left(a_{y}-Avga_{y}\right)}^{2}$$
  (18) $$Vara_{z}=\frac{1}{I_{n}-1}\sum {\left(a_{z}-Avga_{z}\right)}^{2}$$
  (19) $$Vara_{xyz}=\frac{1}{I_{n}-1}\sum {\left(a_{xyz}-Avga_{xyz}\right)}^{2}$$
- Variance of rotational vector in X-direction ($Varr_{x}$), Y-direction ($Varr_{y}$), Z-direction ($Varr_{z}$) and resultant rotational vectors ($Varr_{xyz}$)
  (20) $$Varr_{x}=\frac{1}{I_{n}-1}\sum {\left(r_{x}-Avgr_{x}\right)}^{2}$$
  (21) $$Varr_{y}=\frac{1}{I_{n}-1}\sum {\left(r_{y}-Avgr_{y}\right)}^{2}$$
  (22) $$Varr_{z}=\frac{1}{I_{n}-1}\sum {\left(r_{z}-Avgr_{z}\right)}^{2}$$
  (23) $$Varr_{xyz}=\frac{1}{I_{n}-1}\sum {\left(r_{xyz}-Avgr_{xyz}\right)}^{2}$$
- Signal magnitude area in 2-channels (SMA2) and 3-channels (SMA3) respectively
  (24) $$SMA2=\frac{1}{I_{n}}\sum \left(a_{x}+a_{y}\right)$$
  (25) $$SMA3=\frac{1}{I_{n}}\sum \left(a_{x}+a_{y}+a_{z}\right)$$
- Average of Fourier coefficients of the resultant acceleration ($FFT_{A}$) over kernel length *η*
  (26) $$FFT_{A}=fft\left(\left\{A_{xyz}\right\}\right)$$
- Average of Fourier coefficients of the resultant acceleration ($FFT_{R}$) over kernel length *η*
  (27) $$FFT_{R}=fft\left(\left\{R_{xyz}\right\}\right)$$
- Number of zero crossings along in linear acceleration over *η* in X-direction ($za_{x}$), Y-direction ($za_{y}$), Z-direction ($za_{z}$)
- Average speed (AvgV) and 95th percentile of maximum speed (MaxV)
- **Correlation of linear acceleration in X-Y direction ($corr_{xy}$), Y-Z direction ($corr_{yz}$) and X-Z direction ($corr_{zx}$)**
- Entropy of resultant rotational vector (ER) and linear acceleration (EA) based on normalized power spectrum density (PSD) of resultant rotational vectors ($p_{ri}$) in the time domain and normalized PSD of resultant acceleration ($p_{Ai}$)
  (28) $$ER=\sum -p_{ri}\log_{2}p_{ri}$$
  (29) $$EA=\sum -p_{Ai}\log_{2}p_{Ai}$$
- Average spatial proximity (Euclidean distance) to the bus network (avgBusProx), tram network (avgTramProx), train network (avgTrainProx), street network (avgStreetProx)

Table 1 gives an overview of the different features that are used in different contexts to detect the activity states over atomic segments that leads to trip detection after further merging and refinement.

## 5. Evaluation

In order to evaluate the developed approach, two different types of data sets have been used, each on one context. Data set 1 consists of low frequency sensor data including GPS and IMU sensor signals. Data set 2 consists of a high frequency IMU signal without location information.

### 5.1. Context 1: Availability of Location and Speed Information Along with IMU Signals Sampled at a Coarser Granularity

In the first context, a low frequency (1 Hz, 2 Hz) sensor trace containing GPS and IMU has been used. This is the typical context of smartphone based travel surveys, which generally sample at a low frequency to preserve battery power, as well as to capture real life kinematic behavior during signal loss and in urban canyons.

Since Data set 1 contains GPS points, this data set is well suited for testing also the two existing methods (*walking-based* and *clustering-based*). In the subsequent section a comparative study is performed showing how the three methods perform on the same data set.

#### 5.1.1. Data Set 1: Low Frequency GPS and IMU Data

**Figure 7:**
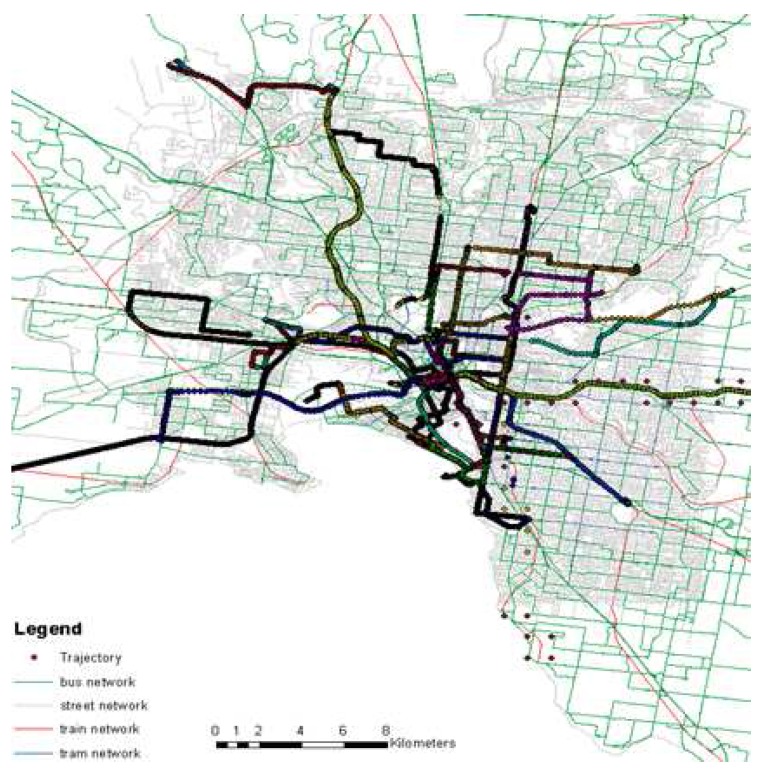
Data set 1: Low frequency (1Hz, 2Hz) GPS trajectories in Greater Melbourne.

The data set (Figure 7) covers different travels in Greater Melbourne, Australia, over three months. The data set contains 0.6 million GPS points over approximately 85 h collected by an Android-based application on a smartphone. The data set reflects different trip behavior in terms of kinematic profile and data quality mediated by four transport modes, *walk*, *bus*, *train* and *tram*, collected between 7 a.m. to 11 p.m. along different routes. In order to capture the real world problems, the data set also covers cases of overlapping routes of different modes and single modes with different speed profiles (e.g., a bus moving *slowly* in the CBD and *fast* along an expressway, whereas maintaining a *moderate* speed in the suburb).

**Figure 8:**
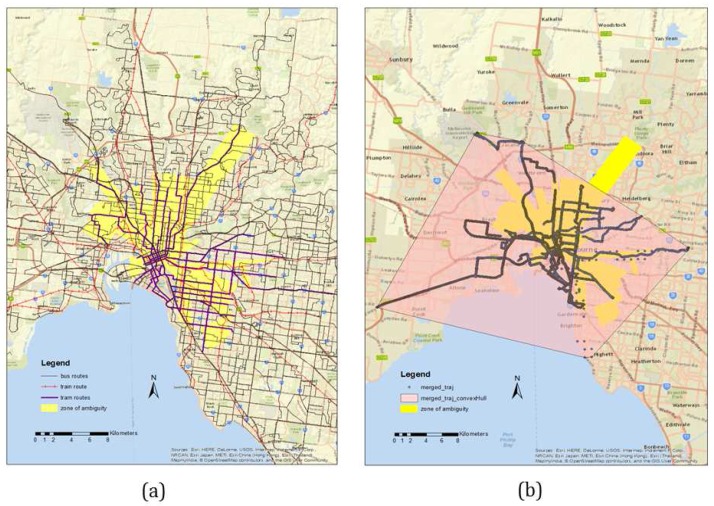
Map (**a**) shows the zone of ambiguity with a significant overlap between different public transport routes; Map (**b**) shows the overlap between the convex hull of the trajectory data set (Data set 1) and the zone of ambiguity.

Since different transport networks frequently overlap, it is often difficult to distinguish between different modes from GPS-only data points. The problem becomes more challenging when there is frequent GPS signal loss or high positional uncertainty due to multipath effects. In order to estimate the overlapping area by the present transport networks (bus, train, tram) a spatial analysis is performed where a set of minimum bounding rectangles (MBR) is developed that contains a given set of route networks. Then an intersection operation is performed to extract the common region that shows a significant overlap by all the public transport route networks. This region is called zone of ambiguity (ZA), which is around 222 sq km (Figure 8a). Another spatial operation is performed on the trajectory data set to generate a convex hull to estimate an extent of the area covered by the trajectories for this experiment (Figure 8b). It is estimated that the data set collected for this research covers 139 sq km of the ZA, which is approximately 63% of the total ZA (Figure 8a).

The data set has been pre-processed using a spatial filter that removes any noise point where the positional inaccuracy is more than 40 m. The data set is collected in the WGS84 coordinate system, which is then projected on to GDA94 zone 55 reference system in order to perform spatial computation on the trajectories in an Euclidean space.

#### 5.1.2. Experimental Setup and Results

In Context 1, two different scenarios are tested. In the first scenario, the full sensor trace is used (GPS and IMU), whereas in the second scenario investigates how the model behaves with GPS only signal and how the accuracy improves when the semantic gap created by the signal loss is bridged by a set of IMU signals sampled at a coarser granularity. A GPS feed sampled at a frequency of ≥1 Hz is state-of-the-art practice in smartphone based travel surveys [3,8,34] and various location based context-aware service provisions [10]. Hence, although the IMU is sampled at a lower frequency, the framework is able to detect the trips and the transfers in between the trips effectively. A prior study on stop detection from smartphone-based travel surveys that also includes GSM trajectories and 3-axis accelerometer signals sampled at a lower frequency demonstrates the efficacy of such sampling strategy [65]. The sampling rate is sufficient for trip or transfer detection, which are phenomena of significantly coarser temporal granularity.

In order to evaluate the framework, 56 trajectories are used as the training sample and 49 trajectories are used as the testing sample for both the scenarios in Context 1. The experiments are realized in three stages. In the first stage (LAYER 1) an atomic kernel of time length *η* is run over each trajectory to generate atomic segments. Each atomic segment is then used to compute a number of features to train a predictive model in Layer 1. In this stage, a near-real time mode detection is performed in order to infer the given activity state. In the second stage (LAYER 2) the atomic segments are merged based on a rule base, where the primary goal is to merge the consecutive atomic segments that bear the same activity state (see Section 4.3.2). This stage generates potential predicted trips. In the third stage (LAYER 3) the predicted trips are further refined based on GTFS information and the lemmata. The basic assumption behind such modular approach is the higher the consistency in mode detection accuracy in Layer 1 the better the inference performance for trip detection in Layer 3.

In order to select the best predictive model in terms of average accuracy and consistency in Layer 1 six different machine learning based classifiers are constructed through supervised learning: a decision tree (DT), a multi-layered perceptron artificial neural network (MLP), a random forest (RF), a K-nearest neighbor (KNN), a naive Bayes (NB) and an ensembled meta classifier (EC-Voting) that predicts based on majority voting by combining three learning algorithms together (e.g., RF, KNN and MLP) to construct the meta-classifier. These classifiers are tested against each test trajectory separately. The classifiers are chosen based on prior studies on transport mode detection and activity recognition. There are ten experiments performed for each of the classifiers using different kernel lengths with different time windows *η*. Table 2 shows the average accuracy of near-real time mode detection for each atomic segment for all the test trajectories in Layer 1 at different *η*.

**Figure 9:**
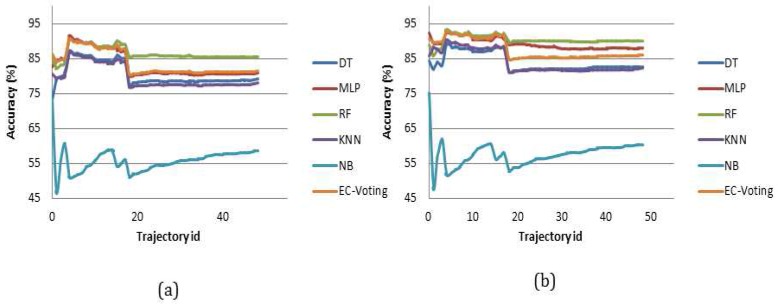
Performance of various classifiers in Layer 1 when using GPS and IMU information at 10 s (**a**) and 60 s (**b**).

Average accuracy reflects the representative measure of each classifier’s prediction accuracy. In order to measure the consistency of the performance of each classifier standard deviation of average accuracy is computed for each kernel length for the same set of test trajectories (Table 3). The result shows a RF based classifier generally yields the maximum accuracy in all the ten experiments with least standard deviation followed by MLP and EC-Voting. A low standard deviation value essentially indicates high consistency with less variation in the accuracy value. However, the difference in average accuracy between a RF based classifier and an MLP based classifier (Table 2) is less. In order to evaluate the statistical significance of their performance measure a paired *t*-test is performed using the individual prediction accuracy made on all the test trajectories. It shows the difference in performance between RF and MLP is statistically significant in eight experimental setups (from 10 s to 60 s and then from 240 s to 300 s) out of 10 experiments (Table 4). This draws a clear contrast between a RF based and MLP based classifier. The result also suggests a RF based classifier outperforms all other learning algorithms used in this paper to infer the activity state (transport modes) in near-real time when using GPS and IMU signal to generate the feature vectors. Figure 9 shows how six different classifiers perform at 10 s and 60 s kernel lengths on all the test trajectories.

In Context 1 Scenario 2 when a sensor trace consists of GPS only information, the performance of different classifiers are evaluated at different kernel lengths. The result shows an MLP based classifier outperforms an RF based classifier in terms of average accuracy (Table 5). In terms of consistency of performance MLP and RF based classifier behave close to each other, however the average accuracy of a RF based classifier is less than that of an MLP based classifier (Table 6). The result also demonstrates that the difference in performance of RF and MLP is statistically significant (Table 7) in nine experiments, except the 240 s window.

Once the atomic segments are generated with a given activity state (transport mode) a rule-based merging operation is performed to generate a set of homogeneous segments for each queried trajectory. Then a pair of stops is retrieved using a ring buffer corresponding to the beginning and ending of the segment. Following that, the segments are now transformed to potential predicted trips with trip start and end in space-time with their stops. These trips are then fed to the Layer 3, where a spatio-temporal consistency check is performed and a refinement process takes place which generates the final trips with their start time and end time, start stop and end stop along with the given transport mode taken during that trip.

For validation purposes in Context 1, the final predicted trips are compared with the reported trips based on trip start time, end time, and the mode. The origin and destination is not validated in this research as the reported trips did not have the complete origin-destination information, but a detailed information on trip start time, end time, and transport mode. However, the framework has a provision to validate the origin, destination and route information if needed (or if the reported data incorporates such detailed ground truth data).

Since there is a temporal uncertainty (Figure 1) associated with the trips due to several reasons (from data end, user end, device end, service end, inference end, and the environmental aspects including the noise and signal loss introduced in the data), while validating the final predicted trips against the reported trips two temporal uncertainty bounds ς are used. Table 8 shows at 10 s kernel length MLP outperforms an RF based classifier in terms of trip detection. But with the growing window RF outperforms MLP in terms of precision and recall accuracy both when 0≤ς≤3 and 0≤ς≤4.

**Figure 10:**
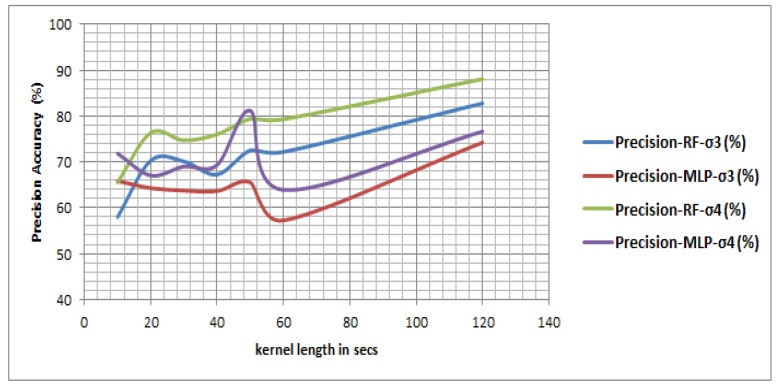
Precision of RF and MLP classifier at different temporal uncertainties.

**Figure 11:**
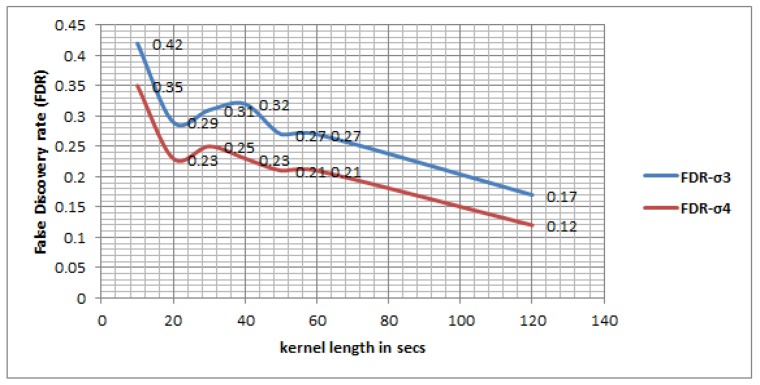
False discovery rate (FDR) of a RF based classifier for trip detection at different ς using a state based bottom up approach.

Figure 10 shows an RF based classifier performs better in general over an MLP classifier—and other classifiers, which is not shown here but evident from the performance in their respective Layer 1 (Table 2). The result shows that with growing upper bounds of temporal uncertainty (ς) the accuracy improves significantly especially for an RF based classifier, where the precision jumps from 57.96% to 65.50% at *η* of 10 s, 70.30% to 76.36% at 20 s, 72.18% to 79.30% at 60 s and 82.78% to 88.07% at 120 s. Table 8 also demonstrates there is a significant improvement in recall when ς is increased from 3 min to 4 min. Figure 11 shows the false discovery rate (thus the Type I error) of a RF based classifier also decreases with growing time window, suggesting that the uncertainty is reduced with growing the kernel length (vis-a-vis the window size). The result also shows when the upper bound of temporal uncertainty is raised from 3 min to 4 min the Type I error has reduced for each kernel length. Since the temporal uncertainty may vary from 3 min to 4 min in this research atomic segments with kernel length of 2 min have been tested assuming there is no change in activity state within that shorter window. The result shows the maximum accuracy is reached at a 120 s window, which is followed by a 60 s window. However, in some situations a quick transfer may take place within 120 s and even within 60 s.

However in Scenario 2, Context 1, in absence of the IMU signal the detection accuracy drops significantly than that of Scenario 1, Context 1. When the sensor trace consists of only GPS based location information without further IMU observations an MLP based classifier performed better in processing Layer 1 as well as in processing Layer 3 and detects trips more accurately than that of a RF based classifier (Table 9). The results in Scenario 1 clearly indicate that although IMU information is sampled at a low frequency this information can bridge the gap present in a GPS trajectory to some extent and helps in detecting the trips. In Scenario 2 using a GPS-only data set the maximum recall and precision obtained using a RF based classifier are 70.77% at 60 s and 64.93% accuracy respectively when 0≤ς≤3, and 79.22% recall at 60 s and 72.72% precision at 120 s when 0≤ς≤4. On the other hand an MLP based classifier yields 75.32% recall and 72.51% precision at 120 s when 0≤ς≤3 and 83.76% recall at 50 s and 75.01% precision at 120 s when 0≤ς≤4.

**Figure 12:**
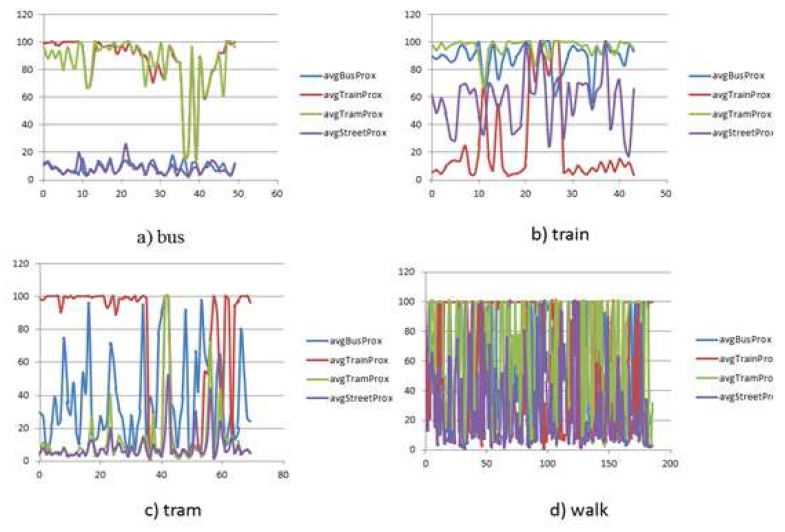
Illustrates average proximity of some of the trips to different route types. Although there is an overlap by the routes of different public transport modes but a trip with a given mode type (for bus (**a**); train (**b**); tram (**c**)) shows a distinct proximity behavior to the given route type. However, since walking can happen anywhere for walking trips there is discernible visual pattern (**d**).

In order to compare with the existing trajectory segmentation and trip detection approaches the data set has also been evaluated using a *walking-based* and *clustering-based* approach. Walking can take place anywhere (along the street, along the train station, close to the bus stop or tram stop) or over any distance (Figure 12). In order to find the most suitable walking distance threshold (L) for the given data set a set of eleven experiments are performed starting with 10 m to 100 m incremented by 10 m, and 200 m separately. The existing walking-based approach segments a trajectory into either a walking or non-walking mode. Thus, for validation purposes any motorized mode is labeled as non-walk. The validation is also performed by measuring the difference between predicted trip start and end time (inferred from *walking based* model) with the reported trip start and end time. Like the state-based bottom-up approach, if the difference for start and end of the trip falls within a given temporal uncertainty then that predicted trip is considered as a true positive.

**Figure 13:**
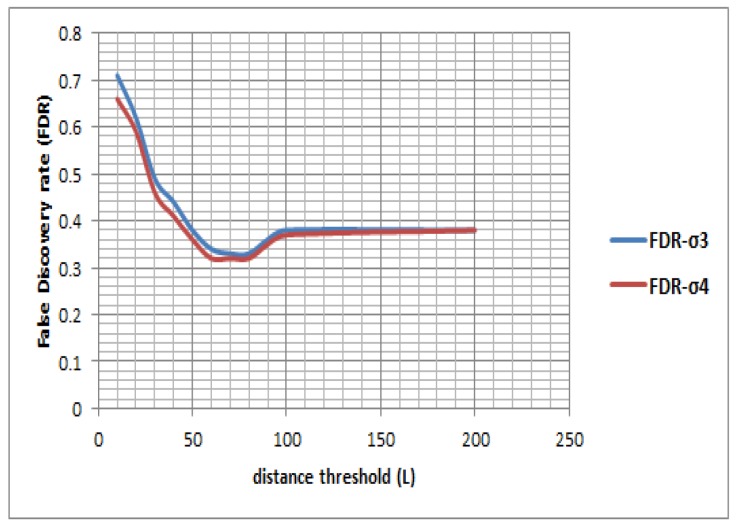
False detection rate (FDR) generated by the walking-based model.

Table 10 shows for the given data set and given movement that behavior maximum accuracy is obtained when the distance threshold (L) ranges from 60 m to 70 m. When ς≤3 the maximum precision accuracy by the walking-based approach is 66.85% and recall accuracy is 75.97% at 70 m distance threshold. The walking-based approach generates many irrelevant segments for shorter distance thresholds, denoting false positive trips when a motorized mode moves very slowly in traffic. On the other hand a longer distance threshold would miss some true positive walking trips thus reduction in precision accuracy. However, in this experiment we have started with a very short distance threshold—10 m to 100 m. Prior studies found a critical distance threshold (>100 m) for effective trajectory segmentation in cities like Beijing [10]. The result shows a shorter distance threshold tends to over-segment the trajectory and gives rise to high FDR and thus to reducing the accuracy of the model (Figure 13). When comparing between the proposed state-based bottom-up model and the walking-based model, it is evident that a walking-based model generates high Type I error owing to high FDR (Figure 11 and Figure 13). For a state-based bottom-up model the maximum FDR obtained is 0.42 (at ς≤4) and the minimum is 0.12 (at ς≤3) (see Figure 11), whereas for walking-based model the maximum FDR is 0.71 and the minimum is 0.32. Both the max-min FDR generated by walking-based approach is higher than the state-based bottom up model. Thus, it is clear the proposed approach is less context-sensitive and less subjective and can work in any environment with a diverse topology of different region of interest (say, transfers between a train stop to the nearest bus stop may be different between cities, which is difficult to model by a walking-based approach but can be effectively detected by the proposed method in this paper).

**Figure 14:**
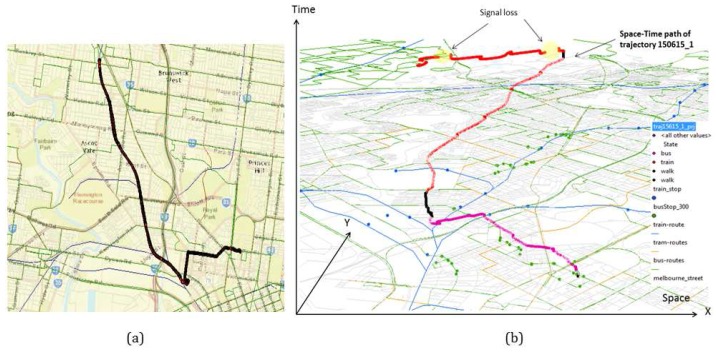
A raw trajectory ID 150615_1 in 2D without any semantic information (**a**); and in 3D as a space-time path with semantic information such as different trips with their start and end in space-time, modes used, travel direction, signal gap, and travel speed (**b**).

**Figure 15:**
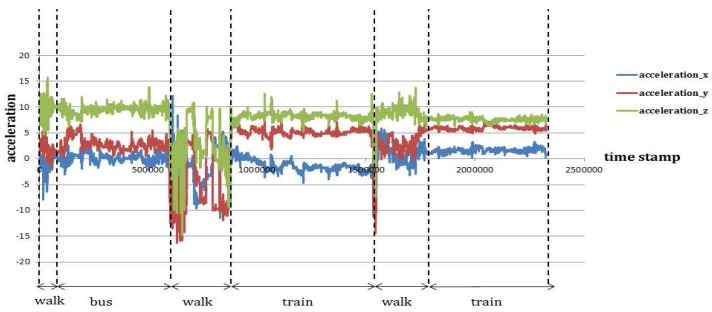
A continuous acceleration profile showing distinct behavior of different transport modes even through the semantic gap due to GPS signal loss on the (TrajectoryID150615_1).

For illustration a test trajectory and its inference process is explained in Figure 14. Table 11 presents a comparison between the reported trips and the predicted trips generated by an RF classifier with 60 s kernel length on trajectory ID 150615_1. Using a state-based bottom-up approach 6 out of 6 trips are correctly detected with trip start and end time and respective transport modes. Using the walking-based method only 4 out of 6 trips are detected, and with less detailed mode information (Table 12). The raw trajectory is shown in 2D; except location information no other semantics is known (Figure 14a), whereas in Figure 14b the same trajectory is shown in 3D in the form of a space-time path with inferred semantics such as different trips with different modes, trip start and end in space and time. In that figure (Figure 14b) the *X*-*Y* space denotes the geographical space and *Z* the time. From the space-time path it is also evident that there are two semantic gaps in the trajectory due to signal loss. The existing approaches such as the walking-based or the clustering-based approach tend to generate misleading information within such gaps. However, the state-based approach bridges these gaps since it is is able to handle IMU information. The IMU signals show a distinct kinematic behavior for the different modes (Figure 15).

The data set is also tested with a clustering-based approach. In this paper we have developed a clustering-based model that produces the geometric clusters of points based on the spatial proximity between the GPS points. The clusters are not semantically enriched. Based on the neighborhood (*ϵ*) and the dwell time (*ϕ*) over each cluster three sets of experiments (based on *ϕ*) are performed where each of the sets contains further ten sets of setups (based on *ϵ*). The minimum number of neighbor points are considered as three, so the total number of points to form a cluster is the core point itself and at least three neighbors. The value of *ϵ* is chosen from 1 m to 10 m assuming the GPS inaccuracy will vary from 1 m to 10 m and beyond in the urban environment using cheap commercial smartphone GPS receivers. However, in real world applications where the location information comes only from GPS feeds (without other location sources such as GSM, Wi-Fi, checkpoints installed in the environment, or from social media) and without semantic enrichment of the clusters, it is evident that the temporal uncertainty is quite high between the predicted trips and the reported trips leading to a low accuracy for all the clustering based experiments (Table 13). The result shows that using a clustering-based method without any semantic enrichment (i.e., without considering the intersected point of interest or other contextual information) a state-based approach outperforms a clustering-based model.

### 5.2. Context 2: Fine Granular Inertial Sensor Information in Unknown Location

Existing approaches (walking-based and clustering-based) rely on the consistent availability of location information. But GPS signal is not available everywhere and also GPS receiver on the smartphone draws on significant amount of energy, hence in Context 2 it has been investigated how the proposed state-based bottom-up approach behaves without location information. It turns out to be adaptive to different contexts, while the existing approaches are not applicable due to lack of location and speed information. Context 2 is also applicable to public health research where one needs to know the current activity state of the user at an even finer granularity (including body movements). As the location information is unknown and a normal body movement frequency is generally 20 Hz [50,66] the sampling frequency is chosen as 50 Hz (which is roughly double of 20 Hz) which also aligns with the prior studies in intelligent transportation systems [50]. Such a high frequency is required mainly due to the lack of location information in the sensor trace. The inference process will solely rely on the IMU signals.

#### 5.2.1. Data Set 2: High Frequency IMU Only Data

In order to evaluate the framework in absence of location information, a high frequency IMU only (linear accelerometer and gyroscope) information is collected across Greater Melbourne sampled at 50 Hz over approximately 8.5 h that covers bus, train, tram and walk trips. Most of the prior transport mode detection research that used IMU signals did not attempt to distinguish between different motor modes [50,54], and only detected pedestrian modes and motor modes. Prior studies also used additionally speed information using a GPS receiver. Here, the inference process is solely based on accelerometer and gyroscope.

#### 5.2.2. Experimental Setup and Results

**Figure 16:**
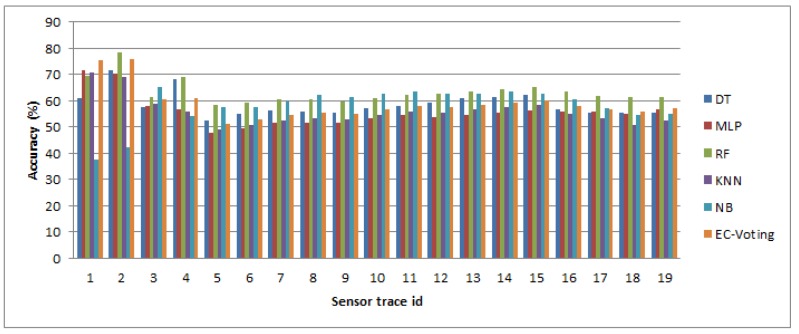
Transport mode detection accuracy using a 5 s kernel over different test sensor traces.

In order to detect the trips using only high frequency IMU data, nine sensor traces are used as training data and nineteen sensor traces are used as testing data. The experiments are performed in two setups. In the first setup a 5 s kernel is run over each sensor trace, and feature vectors are computed from the extracted atomic sensor segments with 50% overlap after passing the atomic segments into a first order low pass filter (LPF) in order to remove any sudden jerk or noise. In the second setup a 10 s kernel is used to generate the feature vectors. In order to avoid the correlation effect (and thus the overfitting of the model) training and testing sensor traces are used separately. The result shows that without using speed information, a sensor trace containing only accelerometer and gyroscope cannot yield very high accuracy (which is in line with a prior research [67]) due to the ambiguity in annotation. For example, a reported tram trip with its trip start and end may have several waiting events in between (at stops), which may not be reported and thus can be misclassified. Also, the vibration of trams and trains may produce similar effects, especially when the train and tram move at a similar speed. During walking changes of speed can happen more abruptly compared to other modes of transport, and thus there is a sharp distinction between walk and non-walk modes in their acceleration profile. Figure 16 shows the accuracy in processing Layer 1 for mode detection on IMU sensor traces. The result shows an RF classifier generally works better than other classifiers and yields accuracies from 60% to 78%. In order to train different classifiers a total 2285 feature vectors are used, whereas a separate set of feature vectors are used for each of the test sensor traces to infer the trips for each of those testing sensor traces. The number of feature vectors ranges from 190 (very short sensor trace) to 1571 .

In Context 2, once the activity states (transport modes) are detected, the atomic sensor segments are fed to Layer 2 where a rule based advanced merging is performed and potential predicted trips are generated. Since in this case the location information is missing, the predicted trips are not further fed to Layer 3 for location consistency checking. Rather the predicted trips generated by Layer 2 are treated as the final predicted trips. Since there is no consistency check hence there are ambiguities in detecting motorized modes, however the models can correctly distinguish between a walking and non-walking mode (bus, train, tram). Hence during validation for the trips generated by IMU only sensor trace, only the predicted trip start and trip end time is matched with the reported trip excluding the activity state (transport mode) at a given temporal uncertainty. When 0≤ς≤3 the recall accuracy for trip detection is 71.05% and precision accuracy is 67.50% using a 5 s kernel.

## 6. Discussion

In this paper a novel state-based bottom-up framework is proposed that can interpret a raw sensor trace and can generate an automated travel diary containing a rich travel information from smartphone based sensor information. A travel diary generated through this framework contains the number of trips, their start and end time, and the particular transport mode used during that trip(s). The model presented in this paper is adaptive and modular in nature. The model is adaptive because it can be applied in different contexts with different types of sensor data and different granularity. The model can generate the activity state information based on a user defined kernel length. The model consists of three phases: an input phase, a processing phase and an output phase. The core of this model is the processing phase which consists of three layers. Depending on the situation each of the layers can be activated or deactivated. For example, if the interest lies in near-real time activity detection (transport mode in this case) then the Layer 1 will be activated and the subsequent layers (Layer 2 and Layer 3) can be deactivated. On the other hand, if one is interested in trip detection from GPS trajectory all three layers can be activated. However, if the same task (trip detection) is to be performed based on IMU only then the third layer is no longer required—thus the model can adapt depending on the requirements and workload effectively.

In this paper we have also introduced the concept of temporal uncertainty (ς), while modeling the trips using Allen’s temporal calculus [11]. The upper bound of ς is considered to vary from 3 min to 4 min depending on the observation for this particular research. The quantification of such temporal uncertainty is done from the fuzziness in traveler and driver behavior, uncertainties in hardware performance (sensors and clock), and the uncertainty present in user’s perceptions of activities while reporting the trips. Since in this research the precision used in temporal information on reported trips is limited to minutes and not seconds, there is always an uncertainty of at least 59 s. Thus, the minimum temporal uncertainty that can be improved in future research will be 2 min by shortening the 3 min minimum uncertainty modelled in this research, which can be further improved if a finer temporal precision is available while recording the ground truth.

In order to illustrate the efficacy and performance of the proposed model for trajectory segmentation and trip generation, it has been compared with two state-of-the-art approaches (walking-based and clustering-based). A walking-based approach is subjective and context-sensitive and thus subject to proper functioning in different situations and for different users. The success of a walking-based approach depends on proper selection of walking speed, distance merging threshold and total distance threshold, which are difficult to set. On the other hand a clustering-based approach depends on the minimum number of points to form a potential cluster based on their spatial proximity. A potential cluster can be treated as a stop or slow walking trip depending on the chosen *ϵ*. The relevance of a cluster can be measured based on the dwell time and other contextual information. In this paper, the clusters formed are simple geometric clusters without any semantic enrichment. The clusters can be of any shape and size, thus raising more uncertainty especially when there is frequent signal gap and randomness in GPS locations. Since both the methods work only when there is a consistent location information (say from a GPS feed) with reasonable accuracy they do not perform well in sparse GPS trajectory data (Context 1) and cannot cope without location information (Context 2). However, the state-based bottom-up method presented in this research can incorporate different IMU information, and hence can work in diverse situations with a reasonable accuracy for mode detection as well as trip detection. The proposed model can also work on a low frequency GPS, combination of GPS and IMU signals, and a high frequency IMU only signal. The model can be made more robust and more intelligent by extending the layers in its processing phase to deal with more diverse and challenging situations, for example, detecting trips and modes on a GSM trajectory, which is generally coarser and more uncertain than that of a GPS trajectory depending on the distribution of cell phone towers.

Despite of the richness in mobility-based activity information the proposed model has some limitations. For example, in Layer 3 while performing the consistency checking an alternate possibility checking is missing at this moment, and that is due to the fact that machine learning algorithms cannot generate an alternate prediction in a human understandable format.

We also investigated the optimal kernel length for detecting transport mode in near-real time. The length of kernels ranging from 5 s to 300 s conforms with the prior studies that attempt to detect mobility-based activities from different perspectives [50,55]. The results show that an RF-based classifier performs better than the other classifiers, and an optimal kernel length can be 60 s to 120 s. However, since some activities, e.g., a transfer, can take place within a 120 s interval, the kernel length can further be reduced to 10 s with the given accuracy. The experimental results show that the performance of the model drops in high frequency IMU only information. This is because the public transport modes (bus, train and tram) can stop at different locations due to traffic signals, congestion, passenger drop off and pick up. During all these events the traveler was most likely being stationary and sitting (or standing) in the vehicle, and the acceleration profile would show a momentary drop during that period. But while reporting the trips, it is difficult to get such a fine ground truth information including how many times a vehicle stopped during a given trip and why. The reported trip is generally annotated as trip start and end time with origin, destination information with a single trip mode type. Thus, if a reported trip mode is *bus*, all atomic segments of the trip are labeled so, although some of them may be actually stationary. This can cause missclassification as well when the predictive model is wrongly trained and detects some of the stationary atomic segment as *bus* and others as *train* or *tram*. When merging the segments in Layer 2, due to this issue some of the trajectories show unreasonable travel behavior, especially Trip ID 1 to 3 (Table 14), where a *bus* mode has been detected in between two *tram* modes, which is not realistic due to the two reasons: (a) if the trip duration ($|t_{3}-t_{2}|$) is very short that means it was actually a continuation of tram trip, but some portion of that particular tram trip has been wrongly detected as *bus*; (b) For some reason if the given trip (Trip ID: 2) is a bus trip then there has to be two walking trips before and after the bus trip as walking can only connect two motorized (or bicycle) modes, which is missing in this case (Table 14).

Such ambiguity can be resolved in a number of ways. In the first approach all the consecutive non-walk trips can be merged together until a discontinuity in activity state occurs or a walk trip is encountered (assuming walking is necessary between two non-walking modes). The first approach is used in this research. However, there may be some cases when a quick transfer may take place shorter than the kernel length, which will generate a Type I error, and wrongly detects a trip with its end time higher than the end time in reported trip. The second approach is collecting even finer ground truth data that should contain intermittent stationary states at different locations (stop, traffic light, congestion, driver fatigue), while traveling in a particular mode in order to train the classifier accordingly. Then while predicting the trips, all the consecutive stationary atomic segments will be merged together until a non-stationary atomic segment is found. The merged segment will be labeled as the immediate non-stationary mode found. However, this approach is tedious, puts cognitive burden on the travelers keeping records for ground truthing, and also deviates these travelers from their normal travel behavior. The third approach can be using a phased sampling strategy (whenever a there is a drop in speed a higher sampling rate can be deployed to record the movement behavior). And lastly the IMU information can be supplemented by speed information from a GPS sensor. Prior studies show using speed information along with the acceleration profile improves mode detection accuracy [54].

## 7. Conclusions and Future Outlook

Understanding travel behavior is important for developing different context-aware services that can enrich mobility as a service (MaaS). Understanding travel behavior is also critical for urban planning and traffic management. Mobility-based activities can also generate information in the interest of public health, analysing a person’s movement behavior at a finer granularity.

In this paper a novel and adaptive state-based bottom-up approach for travel diary generation is proposed, which can detect individual trips with their trip start and end in space and time and the transport mode used to mediate the trip. The approach presented in this paper first detects the activity state on a finer segment (which is called an atomic segment) and then progressively models the trips based on the consistency in the activity state. The reasoning process incorporates a set of machine learning algorithms, heuristic rules and transit feed information. The model is also compared with existing approaches.

In order to test the model, three situations were evaluated using two different real word data sets. The model shows that an RF-based model outperforms other machine learning models in the presence of GPS and IMU information with 0.75 F1-score at 0≤ς≤3 and 0.82 F1-score at 0≤ς≤4 using a 60 s kernel length. On the contrary an MLP-based model works better compared to an RF-based model in absence of IMU information but with a low frequency GPS information, yielding 72.72% and 81.81% recall accuracy at 0≤ς≤3 and 0≤ς≤4 respectively. The model also demonstrates its efficacy in a high frequency IMU only context in absence of location information with accepted loss in granularity in trip information (missing or ambiguous trip mode type). The model also contributes to the knowledge in travel behavior analysis by modeling different types of trips possible at an abstract level (such as actual trip, reported trip, predicted trip and scheduled trip) with their different level of granularity. The results show the proposed model performs better in different situations on different types of data. The model works well even when the existing approaches completely fail especially in absence of location information. The model can also detect a return travel and its direction (Figure 14).

Future research will investigate the notion of alternate solutions in Layer 1. The model also can be improved by more intelligent reasoning schemes to be incorporated during merging operations and consistency checking, such as introducing the longest common subsequence strategy while matching the stop behavior. The model can also be strengthened by implementing a phased sampling strategy to detect finer mobility based activity states especially in the absence of location information. The core of the framework developed in this paper is a hybrid approach which is based on machine learning and a set of heuristics, which can be further enhanced by introducing a further clustering concept whenever there is a consistent location information. Future research can also test other contexts such as on a trajectory with the location information with varied accuracy (e.g., when the source is not only a GPS but also GSM and Wi-Fi).

## Figures and Tables

**Table 1:** Feature selection in different contexts. A ● denotes the corresponding feature is selected under the given context. Likewise ○ denotes the feature is not selected.

FID	Feature	Context 1 Scenario 1	Context 1 Scenario 2	Context 2
1	$Avga_{x}$	●	○	●
2	$Avga_{y}$	●	○	●
3	$Avga_{z}$	●	○	●
4	$Avga_{xyz}$	●	○	●
5	$Avgr_{x}$	●	○	●
6	$Avgr_{y}$	●	○	●
7	$Avgr_{z}$	●	○	●
8	$AvgR_{xyz}$	●	○	●
9	$Vara_{x}$	●	○	●
10	$Vara_{y}$	●	○	●
11	$Vara_{z}$	●	○	●
12	$Vara_{xyz}$	●	○	●
13	$Varr_{x}$	●	○	●
14	$Varr_{y}$	●	○	●
15	$Varr_{z}$	●	○	●
16	$VarR_{xyz}$	●	○	●
17	$FFT_{A}$	●	○	●
18	$FFT_{R}$	●	○	●
19	SMA2	●	○	●
20	SMA3	●	○	●
21	$za_{x}$	○	○	●
22	$za_{y}$	○	○	●
23	$za_{z}$	○	○	●
24	$corr_{xy}$	○	○	●
25	$corr_{xz}$	○	○	●
26	$corr_{yz}$	○	○	●
27	EA	●	○	●
28	ER	●	○	●
29	AvgV	●	●	○
30	MaxV	●	●	○
31	avgBusProx	●	●	○
32	avgTramProx	●	●	○
33	avgTrainProx	●	●	○
34	avgStreetProx	●	●	○

**Table 2:** Average accuracy (%) in Layer 1 for near-real time mode detection using GPS, inertial measuring units (IMU) and spatial information.

	Kernel Length *η* in s									
Classifier	10	20	30	40	50	60	120	180	240	300
**DT**	80.47	83.11	83.48	84.19	83.67	83.91	91.01	90.93	90.63	89.72
**MLP**	83.25	85.25	85.21	88.04	85.55	89.21	92.32	93.25	91.05	91.13
**RF**	86.47	87.53	88.42	89.67	90.12	90.37	92.48	93.48	93.94	93.83
**KNN**	79.74	81.36	82.11	82.25	83.21	84.09	86.03	86.92	86.08	86.92
**NB**	55.84	56.08	57.89	57.69	59.61	57.57	59.87	60.59	62.63	63.58
**EC-Voting**	83.61	85.73	86.01	86.79	86.85	87.55	92.51	93.41	91.04	91.95

**Table 3:** Measure of standard deviation at different kernel length by different classifiers in Context 1, Scenario 1.

	Kernel Length *η* in s									
Classifier	10	20	30	40	50	60	120	180	240	300
**DT**	3.31	2.8	1.76	1.66	2.73	2.59	0.61	1.05	0.81	2.65
**MLP**	3.79	3.12	2.73	1.83	3.58	1.53	0.88	1.27	2.1	0.75
**RF**	**1.88**	**1.72**	**1.61**	**1.48**	**1.51**	**1.29**	**0.86**	**0.72**	**0.71**	**0.74**
**KNN**	3.34	3.22	3.28	3.36	3.13	3.27	2.85	2.74	2.57	1.83
**NB**	3.67	3.72	3.48	3.62	3.63	3.72	3.71	4.18	4.26	3.46
**EC-Voting**	3.51	3.24	3.05	2.84	3.02	2.85	0.63	0.82	2.27	0.67

**Table 4:** Context 1, Scenario 1: Measuring statistical significance of prediction accuracy between MLP and RF based classifiers at 5% significance level. A *p*-value ≤0.05 and *h*-value = 1 signifies the result is statistically significant.

Kernel Length in s	*p*-Value	h
**10**	6.65 × $10^{-7}$	1
**20**	2.14 × $10^{-5}$	1
**30**	2.55 × $10^{-10}$	1
**40**	5.37 × $10^{-6}$	1
**50**	9.50 × $10^{-13}$	1
**60**	9.74 × $10^{-5}$	1
**120**	3.5 × $10^{-1}$	0
**180**	2.8 × $10^{-1}$	0
**240**	1.18 × $10^{-14}$	1
**300**	4.33 × $10^{32}$	1

**Table 5:** Average accuracy (%) in Layer 1 for near-real time mode detection using GPS and spatial information.

	Kernel Length *η* in s									
Classifier	10	20	30	40	50	60	120	180	240	300
**DT**	77.81	83.38	85.02	85.24	81.46	84.45	89.61	91.21	91.54	90.85
**MLP**	82.99	84.36	87.38	87.28	89.15	90.28	93.03	93.37	92.91	91.86
**RF**	76.95	79.12	84.62	85.82	87.13	89.24	91.07	92.69	93.01	92.71
**KNN**	75.26	77.51	78.75	79.02	84.71	86.43	89.05	86.21	90.28	89.61
**NB**	76.07	81.93	83.93	84.88	85.67	86.62	90.76	92.56	92.69	92.76
**EC-Voting**	77.86	79.65	81.78	82.36	87.28	89.05	91.83	93.16	93.65	92.52

**Table 6:** Standard deviations at different kernel lengths by different classifiers in Context 1, Scenario 2.

	Kernel Length *η* in s									
Classifier	10	20	30	40	50	60	120	180	240	300
**DT**	4.11	1.59	1.31	1.19	4.41	3.81	1.07	1.11	0.79	1.05
**MLP**	2.08	1.27	1.53	1.84	1.26	1.35	1.26	1.08	0.84	0.84
**RF**	4.55	4.55	1.28	1.11	1.01	1.21	0.86	0.97	0.79	0.77
**KNN**	4.18	3.93	4.02	3.79	1.27	1.51	0.88	3.47	1.53	1.29
**NB**	3.95	2.23	2.12	1.93	1.98	1.96	1.58	1.41	1.01	0.95
**EC-Voting**	4.83	3.93	4.31	3.94	0.87	1.23	0.58	0.69	0.73	0.82

**Table 7:** Context 1, Scenario 2: Statistical significance of prediction accuracy between multi-layered perceptron artificial neural network (MLP) and random forest (RF)-based classifiers at 5% significance level. A *p*-value ≤0.05 and *h*-value = 1 signifies the result is statistically significant.

kernel Length in s	*p*-Value	h
**10**	3.26 × $10^{-13}$	1
**20**	9.22 × $10^{-12}$	1
**30**	8.29 × $10^{-16}$	1
**40**	7.15 × $10^{-6}$	1
**50**	7.05 × $10^{-14}$	1
**60**	1.19 × $10^{-4}$	1
**120**	2.81 × $10^{-14}$	1
**180**	1.50 × $10^{-3}$	1
**240**	4.77 × $10^{-1}$	0
**300**	1.75 × $10^{-6}$	1

**Table 8:** Context 1, Scenario 1: Trip detection accuracy by RF and MLP based classifier using GPS, IMU and spatial information.

**Classifier: RF**	0≤ς≤3			0≤ς≤4		
η	**Precision (%)**	**Recall (%)**	**F1-Score**	**Precision (%)**	**Recall (%)**	**F1-Score**
**10**	57.96	59.01	0.58	65.50	65.60	0.65
**20**	70.30	75.32	0.72	76.36	81.81	0.78
**30**	70.10	77.27	0.73	74.70	82.16	0.78
**40**	67.25	74.67	0.71	76.02	84.41	0.79
**50**	72.50	75.32	0.73	79.37	82.46	0.81
**60**	72.18	79.20	0.75	79.30	87.01	0.82
**120**	82.78	81.16	0.81	88.07	86.36	0.87
**Classifier: MLP**	0≤ς≤3			0≤ς≤4		
η	**Precision (%)**	**Recall (%)**	**F1-Score**	**Precision** (%)	**Recall (%)**	**F1-Score**
**10**	65.86	71.42	0.68	71.85	77.90	0.74
**20**	64.28	75.97	0.69	67.03	79.22	0.72
**30**	63.74	70.77	0.67	69.00	76.60	0.72
**40**	63.63	72.72	0.67	69.31	79.22	0.74
**50**	65.53	75.32	0.70	81.16	70.62	0.75
**60**	57.22	66.88	0.61	63.88	74.67	0.68
**120**	74.25	80.51	0.77	76.64	83.11	0.79

**Table 9:** Context 1, Scenario 2: Trip detection accuracy by RF and MLP based classifiers using GPS and spatial information.

**Classifier: RF**	ς≤3			ς≤4		
η	**Precision (%)**	**Recall (%)**	**Type I**	**Precision (%)**	**Recall (%)**	**Type I**
**10**	32.41	38.31	123	44.51	52.59	101
**20**	35.38	44.81	126	47.17	59.74	103
**30**	39.11	51.29	123	48.51	63.63	104
**40**	38.74	48.05	117	48.16	59.74	99
**50**	47.33	57.14	98	59.13	71.42	76
**60**	60.55	70.77	71	67.77	79.22	58
**120**	64.93	64.94	54	72.72	72.72	42
**Classifier: MLP**	ς≤3			ς≤4		
***η***	**Precision (%)**	**Recall (%)**	**Type I**	**Precision (%)**	**Recall (%)**	**Type I**
**10**	40.41	50.64	115	46.11	57.79	104
**20**	46.96	60.38	105	52.52	67.53	94
**30**	52.42	70.12	98	60.19	80.51	82
**40**	49.46	60.38	95	58.51	71.43	78
**50**	60.21	72.72	74	69.35	83.76	57
**60**	62.92	72.72	66	70.78	81.81	52
**120**	72.51	75.32	44	75.01	77.92	40

**Table 10:** Accuracy measure of trip detection by walking-based approach.

	ς≤3			ς≤4		
L (m)	Precision (%)	Recall (%)	F1-Score	Precision (%)	Recall (%)	F1-Score
**10**	28.98	51.94	0.37	33.69	60.38	0.43
**20**	37.65	60.38	0.46	40.98	65.58	0.5
**30**	50.47	68.83	0.58	53.33	72.72	0.61
**40**	55.94	73.37	0.63	58.41	76.62	0.66
**50**	61.45	76.62	0.68	63.02	78.57	0.69
**60**	65.53	75.32	0.70	67.23	77.27	0.71
**70**	66.85	75.97	0.71	67.42	76.62	0.71
**80**	66.67	74.02	0.70	67.83	75.32	0.71
**90**	63.58	71.42	0.67	64.73	72.72	0.68
**100**	61.21	65.58	0.63	62.42	66.88	0.64
**200**	61.78	49.35	0.54	61.78	49.35	0.54

**Table 11:** Trip comparison between reported trips and predicted trips in an automated travel diary generated by a state-based bottom-up approach (TrajectoryID150615_1).

Reported				Predicted			
Trip ID	Trip Start	Trip End	Mode	Trip ID	Trip Start	Trip End	Mode
**1**	12:48:00	12:49:00	walk	**1**	12:48:45:412	12:49:43:413	walk
**2**	12:49:00	12:58:00	bus	**2**	12:49:43:413	12:58:10:913	bus
**3**	12:58:00	13:03:00	walk	**3**	12:58:10:913	13:03:00:913	walk
**4**	13:03:00	13:14:00	train	**4**	13:03:00:913	13:14:22:413	train
**5**	13:14:00	13:16:00	walk	**5**	13:14:22:413	13:16:03:912	walk
**6**	13:16:00	13:27:00	train	**6**	13:16:03:912	13:26:56:413	train

**Table 12:** Trips generated by a walking-based method on Trajectory ID 150615_1.

Trip ID	Trip Start	Trip End	Mode
**1**	12:48:16:412	12:49:53:413	walk
**2**	12:49:53:413	12:57:52:912	non-walk
**3**	12:57:52:912	13:03:00:413	walk
**4**	13:03:00:413	13:26:59:412	non-walk

**Table 13:** Trip detection accuracy by a geometric clustering-based model.

**total minPts = (3 + 1) = 4;** ***ϕ* = 60 s**	ς≤3			ς≤4		
ϵ **(m)**	**Precision (%)**	**Recall (%)**	**Type I**	**Precision (%)**	**Recall (%)**	**Type I**
1	48.01	15.58	26	48.01	15.58	26
2	46.15	15.58	28	46.15	15.58	28
3	40.32	16.23	37	40.32	16.23	37
4	44.61	18.83	36	46.15	19.48	35
5	41.42	18.83	41	42.85	19.48	40
6	38.15	18.83	47	40.78	20.12	45
7	36.25	18.83	51	38.75	20.12	49
8	36.71	18.83	50	39.24	20.12	48
9	35.71	19.48	54	38.09	20.78	52
10	34.48	19.48	57	36.78	20.77	55
**total minPts = (3 + 1) = 4;** ϕ **= 120 s**	ς≤3			ς≤4		
ϵ **(m)**	**Precision (%)**	**Recall (%)**	**Type I**	**Precision (%)**	**Recall (%)**	**Type I**
**1**	46.93	14.93	26	46.93	14.93	26
**2**	46.93	14.93	26	46.93	14.93	26
**3**	48.97	15.58	25	48.97	15.58	25
**4**	44.23	14.93	29	44.23	14.93	29
**5**	43.13	14.28	29	43.13	14.28	29
**6**	43.39	14.93	30	43.39	14.93	30
**7**	42.59	14.93	31	42.59	14.93	31
**8**	42.59	14.93	31	42.59	14.93	31
**9**	43.63	15.58	31	43.63	15.58	31
**10**	43.85	16.23	32	43.85	16.23	32

**Table 14:** Unreasonable trips.

Trip ID	Trip Start	Trip End	Mode
1	t1	t2	tram
2	t2	t3	bus
3	t3	t4	tram
4	t4	t5	walk
